# Capillary Electrophoresis Mass Spectrometry: Developments and Applications for Enantioselective Analysis from 2011–2020

**DOI:** 10.3390/molecules27134126

**Published:** 2022-06-27

**Authors:** Shahab A. Shamsi, Ferdoushi Akter

**Affiliations:** Department of Chemistry, Georgia State University, Atlanta, GA 30303, USA; fakter1@gsu.edu

**Keywords:** chiral separations, capillary electrochromatography, electrokinetic chromatography, micellar electrokinetic chromatography, mass spectrometry, chiral selectors, biospecimen, pharmaceuticals, cell cultures, plants and plant extracts

## Abstract

It is now more than 25 years since the first report of enantioselective analysis by capillary electrophoresis-mass spectrometry (CE-MS) appeared. This article reviews the power of chiral CE-MS in resolving issues on the use of chiral selector incompatibility with MS and poor detectability encountered for chiral compounds by UV detection. The review begins with the general principles, requirements, and critical aspects of chiral CE-MS instrumentation. Next, the review provides a survey of MS-compatible chiral selectors (CSs) reported during the past decade, and the key achievements encountered in the time period using these CSs. Within the context of the strategies used to combine CE and MS, special attention is paid to the approaches that feature partial filling technique, counter-migration techniques, and direct use of CS, such as molecular micelles. In particular, the development and application of moving and fixed CS for EKC-MS, MEKC-MS, and CEC-MS demonstrate how various chiral compounds analyses were solved in a simple and elegant way during the 2010–2020 review period. The most noteworthy applications in the determination of chiral compounds are critically examined. The operating analytical conditions are detailed in the Tables, and the authors provide commentary on future trends of chiral separations by CE-MS.

## 1. Introduction

Chiral separations are essential in various analytical applications of pharmaceutical drugs, natural products (including toxins), agrochemicals (pesticides), environmental pollutants, foods and beverages, and biological compounds, such as amino acids, peptides, and sugar, as well as single cells. The last decade has showed significant developments in chiral separations by capillary electrophoresis (CE). In separating chiral compounds where sensitivity is not crucial, ultraviolet/diode array (UV/DAD) is the preferred detection for CE. However, the low sensitivity of the UV detector remains the choking point for trace-level detection of real-life samples. For example, when analyzing metabolites of a parent drug in a complex biofluid, the matrix effect is significant, and the sensitivity observed with UV detection is not sufficient. Laser-induced fluorescence (LIF) detection can be coupled with CE as an alternative to UV for enhanced detection, but LIF can only detect fluorescent compounds. Biological samples that do not fluoresce require an additional labeling step to be detected by LIF.

Because enantiomers have the same molecular weight, i.e., same mass-to-charge ratio (*m*/*z*), their separation is essential before MS detection. The major techniques employed for enantioseparations are high-performance liquid chromatography (HPLC) and gas chromatography (GC). Consequently, hyphenated approaches such as HPLC-mass spectrometry (MS) and GC-MS are mature techniques. Despite the popularity of the HPLC-MS and GC-MS approaches for chiral analysis, they have several drawbacks, including multistep derivatizations and relatively lower separation efficiency. While the recently introduced core-shell particles modified with a chiral selector (CS) in a standard internal diameter (ID) of 4.6 mm provide high efficiency and faster run times in HPLC-MS, these commercially available core-shell chiral columns are expensive. CE combined with MS or tandem mass spectrometry (CE-MS-MS) is a promising alternative to CE-UV due to the increased sensitivity and selectivity, enabling the analysis of chiral compounds in complex samples. Additionally, very low consumption of CS in CE-MS opens up a new possibility for screening exotic chiral selectors and developing a low-cost chiral assay. Several excellent review articles [1,2,3] and book chapters [4,5] have recently summarized the potential of chiral CE-MS. 

The hyphenation of CE and MS has been a developing area of interest for chiral separations due to its high sensitivity, high selectivity, green technology, and low-cost assay. However, our literature search revealed that the number of articles dealing with chiral CE-MS (41 articles) is much lower than chiral CE-UV (549 articles) in the last decade. This trend resonates with the fact that CE, coupled with MS, is still in the developmental stage, and it has many prospects for improvement and significant challenges. Consequently, different solutions are proposed to overcome these challenges. For example, to date, efforts are mainly focused on preventing the contamination of ionization sources by nonvolatile CS with the development of partial filling and counter-migration techniques. Additionally, MS-compatible CS and suitable interfaces are proposed for electrokinetic chromatography (EKC) or micellar electrokinetic chromatography (MEKC). On the other hand, only a few papers reported developing packed and monolithic chiral stationary phases compatible with capillary electrochromatography (CEC)-MS. Thus, in this review, our aim is to identify the number of approaches and avenues for combining chiral CE with MS. Furthermore, we suggest new strategies to promote the development of this hyphenated method.

### 1.1. Identification of Studies in Chiral CE-MS

**Figure 1:**
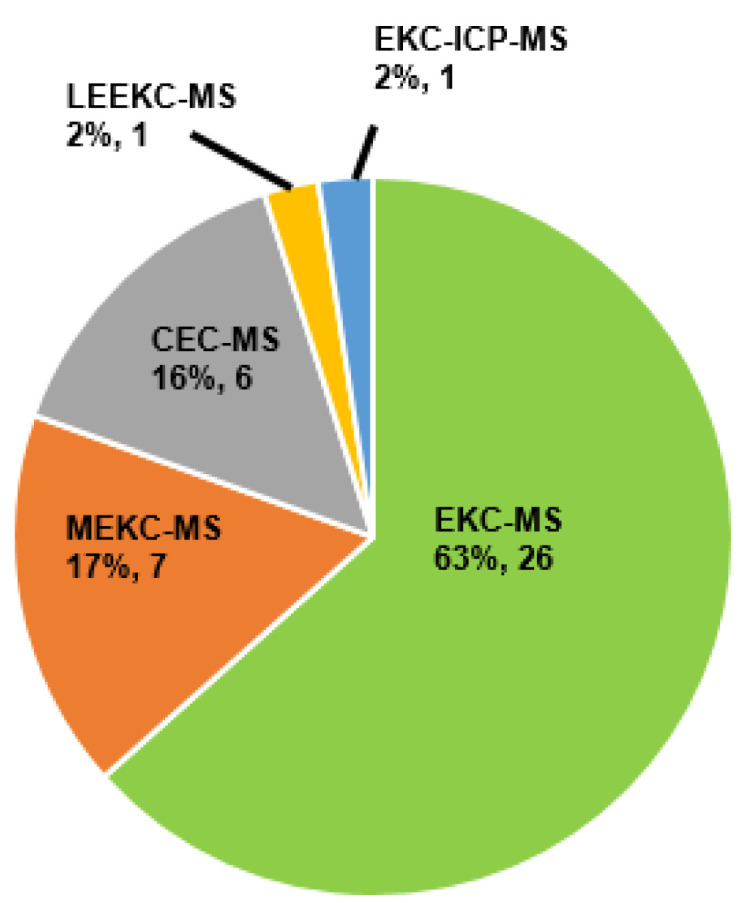
A pie chart illustrates the distribution of articles published on chiral CE-MS modes over the past 10 years (2011–2020).

Figure 1 illustrates a pie-chart distribution of articles published on chiral CE-MS modes during the last 10 years. The CE-MS includes all chiral separation modes [(EKC, MEKC, CEC, and ligand exchange (LE)] coupled to various types of ionization sources (electrospray ionization (ESI) and atmospheric pressure photoionization (APPI)), interfaces (sheath flow and sheathless), and mass analyzers (QQQ, QTOF, and IT). The study and application of three modes of CE-MS ((i.e., EKC-MS, MEKC-MS, and CEC-MS)) for enantioselective analysis have maintained momentum over the 2011–2020 decade, with EKC-MS being the most common working mode. The EKC-MS (63%, 26 articles) is the preferred separation mode, followed by MEKC-MS (17%, 7 articles) and the CEC-MS (16%, 6 articles). The less commonly hyphenated techniques are the EKC-ICP(inductively coupled plasma)-MS (2%, 1 article) and the LEEKC-MS (2%, 1 article). In 1995, Sheppard and coworkers first established the chiral EKC-MS technique by analyzing terbutaline and ephedrine enantiomers using heptakis(2,6-di-O-methyl)-β-cyclodextrin (CD) in low pH buffers [6]. Although the use of molecular micelles (MoMs) (also known as polymeric surfactant) was reported by Wang and Warner in 1994 for chiral MEKC-UV [7], the utility of MoMs for chiral analysis of binaphthol by MEKC-MS was first shown by Shamsi [8]. The chiral CEC-MS version was reported in 1998 at a scientific conference by Mayer to analyze hexobarbital enantiomers [9].

This review presents a comprehensive report focusing mainly on the combined use of CE and MS in determining chiral molecules in various application areas. A search strategy was developed by using keywords such as “chiral capillary electrophoresis-mass spectrometry”, “chiral micellar electrokinetic chromatography-mass spectrometry”, “chiral capillary electrochromatography-mass spectrometry”, and “chiral capillary electrophoresis-inductively coupled plasma mass spectrometry” from four databases (Scifinder, Science Direct, Pubmed, and Web of Science). Certain exclusion factors were followed during the literature search process, and Figure S1 mentions those exclusion criteria. The articles that only deal with chiral separations in GC, HPLC, and SFC combined with or without MS were removed. The items on achiral separations by CE-MS were excluded. Moreover, review articles, book chapters, and dissertations were not considered and articles published in foreign languages were also not included. The citations retrieved from four databases were imported to an endnote to ensure no duplication of items. Recently a review reported on chiral electromigration techniques [1] describes papers published primarily on CE-UV. Only the final section of this review was dedicated to coupling CE to MS-related chiral applications. On the other hand, the present review is focused solely on papers published over the past decade (January 2011–December 2020) on chiral CE-MS. 

A bar chart in Figure S2 compares journals dealing with chiral CE-MS during the review period. The top two journals that published most articles on chiral CE-MS are “*Journal of Chromatography A*” and “*Electrophoresis*”. The miscellaneous category consists of 11 different journals that published one to three articles during the review period. Nevertheless, this bar chart proves that the interest in chiral CE-MS extends to many journals worldwide.

### 1.2. Scope of the Review

This review is divided into several sections. The first sections focus on principles of chiral separation using three CE-MS modes. The second section discusses CE-MS instrumentation, including different interfaces, ionization sources, and MS analyzers. Next, approaches to overcome the CE-MS challenges are discussed in Section 3.4. The third and fourth sections are devoted to using CS for CE-MS and discussing preconcentration techniques to improve CE-MS detectability. The real-world application of chiral CE and CE-MS is mentioned in the last section. Valuable data on the type of chiral analytes separated, sample matrices, BGEs, and chiral selectors used in EKC-MS, MEKC-MS, and CEC-MS are provided in separate tables to make it easier for the reader to search for a specific application. The review ends with a commentary on overall conclusions and future perspectives on the expected developments and chiral CE-MS applications. We believe this review will help stimulate interest in using various modes of CE-MS for analysis and identification of chiral compounds that are often present in biomedical samples with limited volumes, as online CE-MS and microchip CE-MS are ideally suited to investigations of these samples.

## 2. Fundamentals of Electromigration Techniques for Chiral CE-MS

Often, the various electromigration techniques in CE differentiate enantiomers. In contrast, the enantiomers are indistinguishable by MS because they have the same mass and show identical mass spectra, and, without the CS, they cannot be separated. The separation principle of chiral CE is somewhat different and more complex than the conventional CE method. In traditional CE, the charged entities migrate through a narrow capillary column (I.D. as small as 50 µm) under an electric field with different electrophoretic mobility, dependent on the shape, size, and charge density. The conventional CE would fail to separate enantiomers with similar charge densities, and electrophoretic and electroosmotic velocities, thus, making it impossible to resolve the two enantiomers by any non-enantioselective reagent. 

Two different ways have emerged for chiral separations by CE, classified as the indirect and direct mode. The indirect method involves adding a sterically pure chemical to derivatize the racemic compounds. The new derivative pairs in the diastereomeric form migrate based on their physicochemical properties at a different elution order. The less popularity of this indirect approach is due to the limited availability of chiral derivatization reagents. On the other hand, the direct mode introduces a chiral environment, i.e., adding a free-floating CS in the background electrolyte (BGE) or immobilized in the column as the stationary phase. The separation in chiral CE follows both chromatographic and electrophoretic mechanisms. The chromatographic part arises from forming the diastereomeric complex between the CS and the enantiomers. In contrast, the electrophoretic part comes from the slight difference in mobility of the enantiomer-selector complex. Because there are subtle differences in the size of these complexes, one enantiomer could be more or less “trapped” in the structural moiety of the CS. The diastereomeric complex between the enantiomer and the CS is formed by intermolecular interactions, such as ion–ion, ion–dipole or dipole–dipole, π-π, van der Waals, and hydrogen bonds. Steric factors from the spatial arrangement of the cavity of the CS play an essential role in enantioseparations. Apart from the aforementioned enantioselective interactions, the solute’s adsorption to the column walls must be considered to understand the analyte retention behavior. For more recent details on the enantioselective mechanisms in CE, several excellent reviews are available [10,11].

### 2.1. Modes of Chiral CE-MS

In this section, fundamental principles of several modes of chiral CE-MS, such as chiral EKC, MEKC, ligand exchange electrokinetic chromatography (LEKC) using a moving chiral selector, and CEC using a fixed chiral selector, are briefly discussed.

#### Moving Chiral Selector

**Figure 2:**
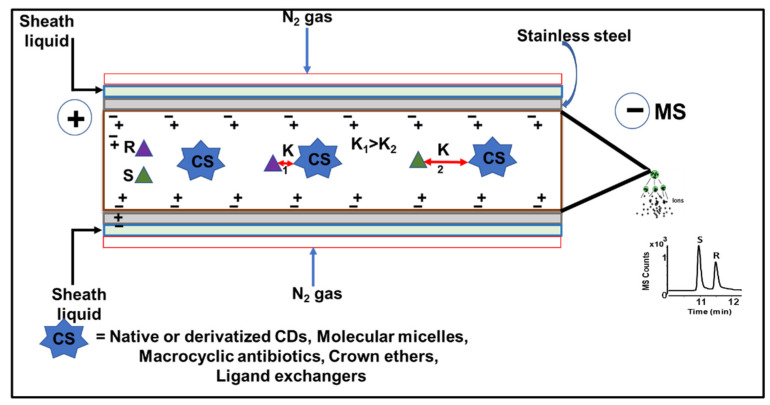
Schematics of chiral CE-MS under positive polarity. The chiral selector can be native cyclodextrins (CDs), derivatized CDs, macrocyclic antibiotics, crown ethers, ligand exchangers, and anionic molecular micelles (MoMs).

In CEKC, CMEKC, and CLEKC, the moving CS, also known as the “pseudostationary phase” (PSP), is dissolved into the BGE, similar to using a stationary phase in a chromatographic column. The chiral analytes retention order differs from the conventional CE because of the differences in the affinity between the charged or neutral chiral analytes with the neutral PSP [12,13]. However, it should be noted that the CS in EKC, MEKC, and LEKC can also be negatively or positively-charged reagents. A schematic representation of moving CS is shown in Figure 2. A sample with two positively-charged enantiomers (*R*- and *S*-) is injected from the capillary inlet end. Upon applying the voltage, the two enantiomers interact with any one of the CS (e.g., native or derivatized CDs, polymeric surfactants (also known as MoMs), macrocyclic antibiotics crown ether, or ligand exchangers) and form diastereomeric complexes with different binding affinities. Because the binding constant of the second enantiomer (K_2_) is greater than the binding constant of the first enantiomer (K_1_), the more complex form moves slower than the less complex form towards the MS detector end. Therefore, in this case, *R*-enantiomer elutes faster than *S*-enantiomer. There are three different categories of moving CS: (a) moderate molecular mass compounds (native, derivatized, and charged CDs, macrocyclic antibiotics, and crown ethers) used in EKC-MS; (b) high molecular mass compounds (e.g., MoMs) in MEKC-MS; (c) ligand exchanger (e.g., copper (II) added to the CD derivative) in LEKC-MS.

### 2.2. Fixed Chiral Selector

**Figure 3:**
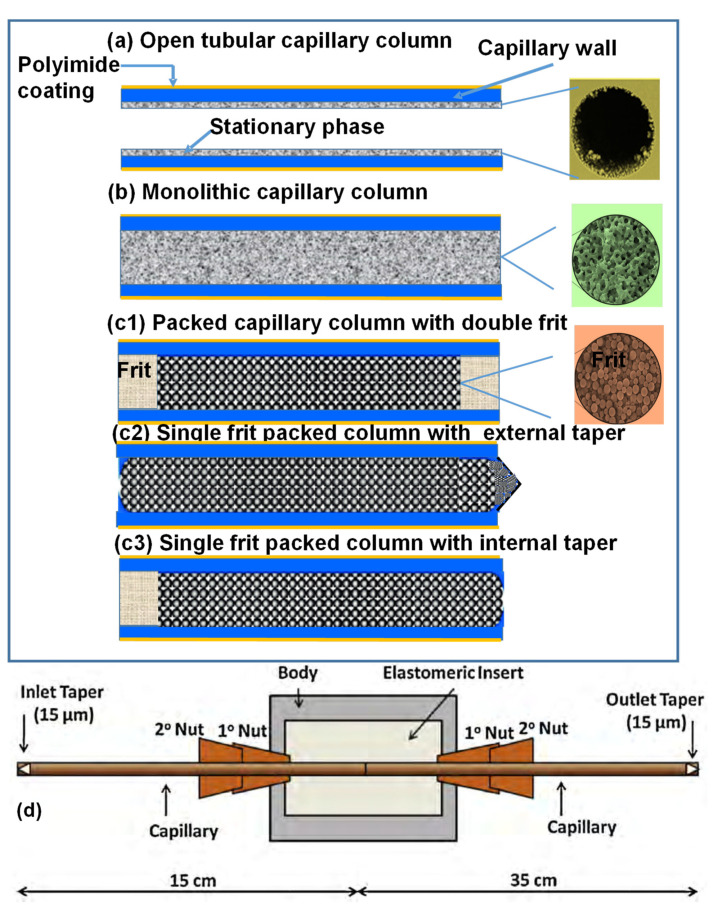
A schematic representation for the types of columns used in chiral CE-MS: (**a**) open tubular column, (**b**) monolithic column, and packed column with (**c1**) double frit; (**c2**) single frit and external taper, and (**c3**) single frit and internal taper; (**d**) fritless column with the new objective picoclear connector. The elastomeric insert of the connector ensures that the two capillaries do not grind together when joined. The arrows at the bottom represent the lengths of the inlet packed portion (15 cm) and outlet packed portion (35 cm) of the fritless column (not to scale). (**a**–**c3**) is reproduced with permission from A. S. M. Mojibur Rahman Chawdhury (dissertation in preparation) at Georgia State University, and (**d**) and Figure caption are adapted from Bragg et al. [15] with permission from Elsevier.

Instead of a moving chiral selector in the BGE, CEC-MS uses a chiral stationary phase (CSP) immobilized on the capillary wall to achieve enantioseparation without causing MS contamination. This hybrid technique of CE and HPLC incorporates an actual CSP as particles, monolithic bed, bonded, or coated material where EOF moves analyte and mobile phase by applying an electric field. Three types of CEC-MS columns are represented in Figure 3: (a) open tubular (OT), (b) monolithic, and (c–d) packed. While open tubular and monolithic columns can easily separate charged and uncharged chiral compounds, several versions of packed chiral columns are reported for CEC-MS, such as double frit (Figure 3c1), single frit with an external taper (Figure 3c2), single frit with internal taper (Figure 3c3) as well as fritless packed column (Figure 3d). The pros and cons of various versions of the packed CEC-MS column are discussed in earlier publications [14,15]. In all three forms of column technology developed for chiral CEC-MS, mobile phase parameters, such as pH, buffer ionic strength, organic solvent, and the applied voltage, influence chiral separations.

The OT-CEC-MS (Figure 3a) is the most easily fabricated and ESI-MS friendly among the three types of columns due to ease of fabrication and stable EOF. Because the type of CSP (adsorbed or covalently bonded dictates the EOF), OT-CEC-MS can conveniently modify the capillary walls with a suitable CS. Wachs and coworkers showed the application of OT-CEC-MS for enantioseparations in 1998 [9]. However, there are zero publications reported for OT-chiral CEC-MS during this review period. Recently, nanomaterials have been suggested as a promising route for CSP synthesis for the above-mentioned mode of CEC [16]. Self-assembly of the chiral nanomaterials on derivatized capillaries can be adopted to develop such OT columns for CEC-MS. 

Monolithic columns in chiral CEC-MS (Figure 3b) have gained some interest due to the flexibility of fabricating stationary phases (silica or polymeric base), fritless design, and large column capacity. However, the chiral monolithic column must have superior porosity and many chiral interaction sites for effective enantioselectivity by CEC-MS. Schweitz and coworkers first introduced molecularly imprinted polymers as a monolithic phase for CEC-UV [17]. However, Gu and Shamsi first demonstrated chiral CEC-MS using a monolithic column [18]. A methacrylate-β-CD monolith showed excellent stability, repeatability of retention time, and enantioselectivity of hexobarbital as a model chiral analyte. Correctly configured monoliths have an additional advantage of handling high liquid flow for CEC-MS due to macropores and mesopores presence within their structure. Furthermore, monoliths offer high permeability, fast mass transfer, high selectivity, and better chromatographic efficiency. More details on the type of monolithic columns used in chiral CEC-MS are presented in Section 4.3.

The bubble formation in packed CEC columns with double frits configuration (Figure 3c1) with UV-detection can be reduced by pressurization of both capillary ends, the same approach is not feasible with MS detection. The chiral separation using packed column CEC-MS with fast atom bombardment for ionization was reported by Verheij et al. in 1991 [19]. After that, Brocke et al. in 2002 [20], as well as Zheng and Shamsi in 2003 [14], reported the use of chiral packed column for CEC-ESI-MS. Using a single frit with external or internal tapers (Figure 3c2,c3, respectively), one end of the capillary is held at atmospheric pressure at the interface that connects the capillary to the MS. In particular, developing a single-frit particle-based column with an internal taper [14] or a fritless packed column [15] are proposed as two viable options for CEC-MS. A novel design of a fritless packed column was proposed using two internal tapered columns joined together by a commercially available picoclear connector (Figure 3d) [15]. The column was packed with 3–5 µm silica particles coated with polysaccharides to form a CSP. Several benefits of fritless packed column include: (a) absence of frits eliminates the possibility of bubble formation, (b) more robust columns with good column-to-column repeatability than external tapered columns, and (c) higher efficiency than an internal tapered column with no dead volume are noted. Nevertheless, utilizing a fritless packed column is a significant step toward developing a repeatable chiral CEC-MS. 

## 3. Basic Instrumentation-Setup 

### 3.1. Leveling the CE and MS Instruments

**Figure 4:**
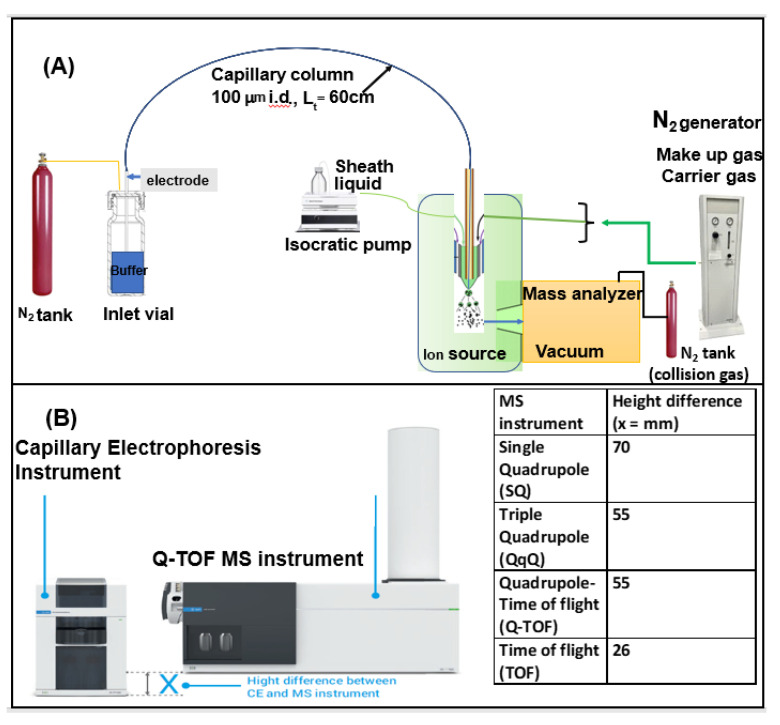
A general illustration of the CE-MS instrument arrangement shows: (**A**) experimental set-up equipped with a sheath liquid pump, an orthogonal ESI source, make-up carrier gas, and collision gas supplied by a nitrogen generator and a nitrogen tank, respectively; (**B**) positioning the bench to adjust the sprayer height above bench level (X). The inset table on the right specifies height adjustments for different mass analyzer types (the graphics are courtesy of Agilent Technologies). (**A**) is reproduced with permission from A. S. M. Mojibur Rahman Chawdhury (dissertation in preparation) at Georgia State University.

The essential CE-MS equipment for chiral analysis consists of a power supply that provides an electric field, one electrode, one volatile electrolyte-filled buffer reservoir, a fused silica capillary (25–100 µm I.D.), an injection module (hydrodynamic or electrokinetic), a nitrogen tank to provide an external high-pressure flush (up to 2 bar) of the capillary with electrolyte solution, a nebulizer (sprayer with three concentric tubes), a sheath liquid pump, ionization source, a mass analyzer, and a data collection system (Figure 4A). When interfacing the CE to the MS system, the two instruments should be placed in close proximity to minimize the total length of the separation capillary connecting them (Figure 4B). One manufacturer recommends placing the CE instrument on a height-adjustable cart simplifies positional adjustment. Additionally, the buffer level in the capillary inlet vial should be at an equal height relative to the buffer level in the nebulizer located at the capillary outlet vial to avoid siphoning; otherwise, a current breakdown will occur if the outlet height at the MS end is below the inlet vial. In some commercial CE instruments, the capillary in the inlet vial terminates 365 mm above the bench level. Adding to this, the buffer fill height of 15 mm provides a total height measurement of 380 mm. Therefore, it is crucial to match the outlet capillary tip in the triple tube sprayer at the same height as the buffer level in the inlet vial. The Agilent 7100 CE instrument has a T-shaped sign on the right panel indicating the electrode position in terms of height, which can serve as a reference for height adjustment. A cart or a table that is lower than the bench height (26–70 mm) on the various types of MS analyzer (Table inset in Figure 4B) and adjustable in height resolves the requirement for leveling. 

**Figure 5:**
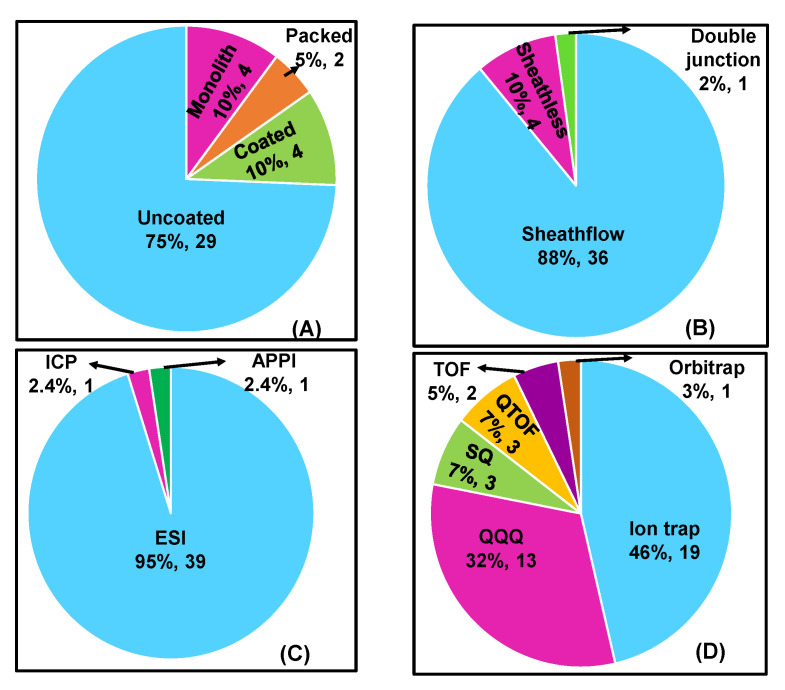
Pie charts showing the distribution of various (**A**) column types, (**B**) interfaces, (**C**) ionization sources, and (**D**) mass analyzers used in chiral CE-MS over the period 2011–2021.

A pie chart in Figure 5A dictates the trend using CEC-MS columns compared to uncoated silica columns in MS for chiral separation. The majority, 75% (29 articles), of the publications, involved uncoated bare silica capillaries, and 10% (4 articles) involved coated/covalently bonded or monolith columns. Only 5% (2 articles) were reported for packed column CEC-MS. The above-mentioned distribution indicates a considerable challenge to utilize monolith, packed, or even coated capillaries for enantioselective CEC-MS. However, it is worth mentioning that once the CEC column is correctly designed with appropriate porosity, permeability, and enantioselective sites, it results in a robust coupling to ESI-MS.

### 3.2. Interface and Ionization Sources 

An interface is a conjunction between the end of a capillary from the CE outlet and the inlet of the MS instrument ionization source. The design of the interface maintains the electrical connection from the capillary tip to the ionization end. Two CE interfaces (sheath and sheathless) are now commercially available. The co-axial sheath flow interface is widely used compared to the sheathless interface. The pie chart in Figure 5B reveals that the majority, 88% (36 articles), of the publications on chiral analysis employed a sheath flow interface compared to only 10% (4 articles) on the sheathless connection. This trend is, perhaps, because sheath flow was commercialized almost 20 years ago, whereas sheathless interface was commercialized later, in 2012. In addition, there is only one report on the use of a commercial double-junction interface for chiral analysis [21].

A triple tube design of a commercially available co-axial sheath flow interface is shown in Figure S3A. In this configuration, the capillary filled with a CS (dissolved in a volatile buffer solution) is inserted through the interface’s central tube, “c”. As mentioned earlier in this section, the central tube is surrounded by a second stainless steel tube, “b”, that delivers sheath liquid to the capillary tip. Volatile solvents are mixed with appropriate acids or bases in a sheath liquid mixture to build sufficient conductivity to complete the CE capillary electric path. The sheath liquid then merges with the analyte solution emerging from the CE capillary. The emerging solution forms a fine spray from the needle end, “d”, by applying a few kilovolts of potential across the spray chamber. Next, the spray is converted to small droplets using a flow of nebulizing gas (N_2_ gas) from a tube, “a”. An electrode grounds the electrical connection, “f”, to create a return path for CE and ESI current. The actual sprayer is represented on the right side of Figure S3A**,** where three concentric stainless-steel tubes are marked as a, b, and c. A pneumatic HPLC pump is connected to the sheath liquid reservoir to deliver sheath liquid to the nebulizer at a typical flow rate of 1–10 µL/min. The sheath flow interface is compatible with different ionization sources, such as ESI, atmospheric pressure photoionization (APPI), and ICP, discussed later in the following paragraph. The sheath flow interface has many advantages, including flexibility of adapting to different ionization sources, availability of different commercial designs for sheath flow, and ionizing a wide range of chiral molecules with a broad range of *m*/*z*. However, one disadvantage of the sheath flow ESI interface is sample dilution that is caused by using sheath liquid, which also creates significantly noisy ESI-MS spectra, resulting in insufficient detection limits. 

Moini et al. tackled this disadvantage of the sheath flow by designing a sheathless CE-MS coupling where non-volatile BGE provides no interference with the MS signal [22]. The set-up is relatively simple and involves only BGE as the conductive liquid. The signal is enhanced as the design does not require sample dilution from the sheath liquid. The sheathless interface fabrication is performed by etching the CE capillary outlet end using hydrofluoric acid, resulting in a porous capillary end. Figure S3B shows that the porous end inserted in a stainless-steel needle filled with a volatile BGE serves as a conductive medium to maintain electrical contact between the needle and the porous capillary tip. The ESI voltage helps spray formation at the porous end to form ions entering the MS analyzer. There are several disadvantages of a sheathless interface. First, the porous tip is not designed to handle large sample flow as the capillary I.D. is restricted to only 30 µm. Second, the porous capillary lifetime extends to as high as 200 injections, which can only be replaced by materials manufactured by a company named Sciex. The consumable cost and limited options for the sheathless interface seem to be the barrier before this type of interface is routinely used.

There are various ionization sources that can be used as the interface between the CE eluent and the mass spectrometer. The three ionization sources used in chiral CE, i.e., ESI, APPI, and ICP, are reported in this review period. The majority, 94% (39 articles), have utilized ESI sources for chiral analysis, one article has been published using ICP and APPI as the ionization source (Figure 5C). Both sheath and sheathless interfaces discussed in the previous section can be coupled with ESI to ionize charged entities from the volatile BGE. The basic setup for spray formation in ESI is illustrated in Figure S3A. Three steps are followed to transfer ions in ESI from solution to the gaseous phase, including (i) formation of the fine spray of charged chiral analyte droplets using sheath flow or porous capillary tip, (ii) nebulization or evaporation of volatile solvents from the spray mixture utilizing a flow of dry nebulizing gas and ESI potential, and (iii) discharge of ions from the nebulized droplets to the gaseous phase.

The soft ionization mode to ionize weakly polar or non-polar neutral chiral compounds is APPI. The principle of APPI-MS is shown in Figure S4, where the CE effluent (containing the injected sample), when combined with a sheath liquid, forms a gaseous analyte (M), which is then ionized by the photochemical action initiated by a krypton discharge lamp emitting photons of ~10.0 eV. Next, the analyte molecules formed after photoionization in the gaseous phase are eventually transferred with voltage to the mass analyzer. Both analyte and sheath liquid solvent are excited during the photochemical ionization, allowing the analyte to acquire protons from the protic solvents. A spacer made of insulated polymer with an adjustable length of ~41 mm is positioned for APPI so that it allows to mount the CE nebulizer on the electrospray chamber. This spacer is commercially available and is compatible with all different types of mass analyzers. There is only one reported publication on the combination of APPI-MS [23] and chiral CE in this review period, even though APPI faces relatively less ion suppression from the matrix than ESI. 

The ICP as an ionization source allows trace analysis of non-volatile chiral components. The successful CE-ICP-MS coupling requires specific criteria to be fulfilled, such as (i) maintaining a steady electrical connection at the end of the CE capillary, (ii) matching the flow rate of the CE capillary (μL/min) with the uptake rate of the ICP-MS (mL/min) to ionize weakly polar or non-polar neutral chiral compounds for the stable operation, and (iii) efficiently introducing analytes from CE capillary to the plasma. The CE-ICP-MS set-up also requires interfaces (sheath or sheathless) to combine CE and MS instruments. Figure S5 shows that CE-ICP-MS is coupled to a sheath-flow interface where a microconcentric nebulizer delivers sheath liquid and nebulizing gas at the interface to form fine droplets. This nebulizer is the same as used in ESI-MS. The nebulized droplets are later transported into the plasma torch of the ICP-MS to convert them into ions. There are two critical advantages of chiral ICP-MS. First, this technique is specifically helpful for chiral metallodrugs [24] (used in chemotherapy treatments), studying chiral metal–ligand interactions, and speciation analysis. Second, unlike CE-ESI-MS, the CE-ICP-MS does not require volatile electrolytes or volatile chiral selectors. 

### 3.3. Types of Mass Analyzers

The mass analyzers differentiate ions in the gaseous phase based on their *m*/*z*. There are many types of mass spectrometers available for interfacing with CE. The distribution of the analyzers used for chiral CE-MS is shown as a pie chart in Figure 5D. Six different mass analyzers adopted for chiral analysis in the past decade are: (i) ion-trap, IT (46%, 19 articles), (ii) triple quadrupole mass analyzer, QQQ (32%, 13 articles), (iii) single quadrupole mass analyzer, SQ (7%, 3 articles), (iv) time of flight, TOF (5%, 2 articles), and (v) quadrupole time of flight mass analyzer, Q-TOF (7%, 3 articles) and (vi) orbitrap (3%, 1 article).

As shown in Figure 5D, one of the most common mass spectrometers used for chiral CE-MS is the IT mass analyzer, which can produce important MS^n^ data when performing structural elucidation assays. In an IT analyzer, ions are subjected to three hyperbolic electrodes in a three-dimensional space under radio frequency (rf) and direct current (dc) voltage. Notably, ions are trapped within the three-electrode system with a stable motion and without any degree of freedom. The trapped ions can be released and scanned over a range of *m*/*z* values last. Single ion monitoring (SIM) is achieved by isolating a specific ion in the trap by applying a voltage. In contrast, multiple reaction monitoring (MRM) occurs when fragments ions are introduced into the trap with an inert gas, such as argon or nitrogen.

The triple quadrupole (QqQ) MS-MS system is the second most commonly used mass analyzer reported in the last decade for chiral analysis by CE-MS. The MRM can be achieved when a single quadrupole (SQ) is combined with an additional quadrupole analyzer and a collision cell. In a QqQ analyzer, the first quad filters a parent or precursor ion before it can be fragmented inside the collision cell (q). The fragmented or the daughter ions formed in the collision cell are low molecular weight ions, structurally similar to the parent or precursor ion. These fragmented ions then enter the final mass analyzer to be monitored and scanned, providing structural ion information.

The SQ analyzer applies rf and dc voltage on four metal rods within a two-dimensional space to scan over different *m*/*z* values (usually < 4000 *m*/*z*) to construct mass spectra. The scanning speed can be increased up to 1000 *m*/*z* per second with accuracy as best as 0.1 *m*/*z*. This is the third most commonly used mass analyzer for chiral CE-MS that will provide a mass spectrum for each peak that elutes from the CE column before being analyzed by the MS system. The SQ analyzer can monitor a single ion in SIM mode by stepping up voltages in a few milliseconds, enhancing a specific analyte detection limit. 

The time-of-flight (TOF) mass analyzer accelerates ions by applying a high potential across the flight tube. The *m*/*z* value dictates how much time is required for an ion to travel the flight tube and reach the detector. A mass spectrum can be constructed by pulsing the accelerating potential and converting the output result as a function of time. The TOF mass analyzer can be coupled to CE to measure the mass of the enantiomeric molecules with high accuracy and enhanced sensitivity. Hybrid mass analyzers combine two basic types of mass analyzers to make a specialty system. One example of a hybrid mass spectrometer is the Q-TOF MS/MS system, which combines a quadrupole mass spectrometer with a TOF mass spectrometer. A tandem Q-TOF/MS-MS instrument is developed by replacing the third quadrupole of a QQQ analyzer with a TOF analyzer to analyze chiral compounds with both high sensitivity and mass accuracy. Unlike conventional ion traps, the orbitrap uses only electrostatic fields to confine and analyze ions. Although orbitrap in LC-MS is one of the new emerging trends, the orbitrap mass analyzer for chiral CE-MS is reported in only one publication [25], but its advantage was not clearly illustrated. We expect this mass spectrometer to be used in CE-MS applications in future studies involving identifying and quantitation of untargeted chiral compounds.

### 3.4. Approaches Used in Coupling Chiral CE to MS

As mentioned earlier, CE-to-MS coupling is not straightforward in chiral analysis using low molecular CS (e.g., native or derivatized CDs, crown ethers, macrocyclic antibiotics, and unpolymerized micelles). It is now well established that the accumulation of these CS mentioned above causes the ionization source fouling, limiting the ESI-MS detection. To overcome these drawbacks, three approaches to improve sensitivity are: (a) partial filling technique (PFT), (b) counter-migration technique (CMT), and (c) use of MoMs.

#### 3.4.1. Partial-Filling Technique

In the PFT, the separation capillary is partially filled with neutral or charged CS. First, the capillary is flushed with BGE without adding CS, followed by injecting a small plug of CS (charged or neutral) containing buffer. Upon injecting the chiral analytes into the capillary, either by hydrodynamic or electrokinetic injection, the enantiomeric mixture enters the CS zone to begin enantioseparation through the partition mechanism (Figure S6A, left). The separated enantiomers then travel towards the MS detector, leaving the plug of CS behind. Hence, the resolved enantiomers enter the MS detector and the buffer flow without contaminating the MS detector with nonvolatile CS. This technique helps minimize ion suppression from low volatile CS in EKC, MEKC, or LE separations. However, one drawback of the partial-filling technique is that it allows only a plug of CS into the CE capillary, compromising the chiral resolution.

#### 3.4.2. Counter-Migration

The CMT utilizes an oppositely charged CS moving opposite to the analyte and the EOF. Chakvetadze et al. introduced the CMT for EKC-UV [26], later extended to EKC-MS by Schulte and co-workers [27]. An example of a counter-migration-based enantioseparation is illustrated for EKC-MS in Figure S6 (right). Using a positive polarity, highly negatively-charged CS (e.g., SBE-β-CD) would have mobility towards the anode, i.e., the injector end. When t = 0, the basic enantiomeric mixture starts migrating towards the cathodic end when injected at the anodic end and comes in contact with the negatively-charged CS at a particular capillary segment (Figure S6B, top right). When t_1_ > 0, the injected analytes interact with the CS, get separated into enantiomeric pairs 1 and 2, and move towards the MS detector end (Figure S6B, middle right). After the enantioseparation occurs, the plug of CS moves away toward the injector end, and the analyte migrates eventually to the MS detector end with the help of EOF. Therefore, it should be noted that highly negatively-charged CS mobility is not overcome by the EOF (i.e., µ_SBE-β-CD_ > µ_EOF_) and, thus, avoiding the entrance of the CS into the MS ionization source when t_2_ > t_1_ (Figure S6B, bottom right).

#### 3.4.3. Molecular Micelles

The development of MoMs is a direct approach to hyphenate chiral CE to MS. An electrospray behavior of low molecular mass (unpolymerized micelle) versus high molecular mass is shown in Figure S7a. The unpolymerized micelle dissociates into surfactant monomers causing significant background noise, whereas MoMs, being covalently stabilized, remain undissociated in the ESI-MS (Figure S7b). There are several advantages indicated by Shamsi [8]. First, the MoMs, with their high molecular weight and fixed molecular structure, can sustain ESI ionization, and, thus, MS signal for the analyte is not significantly suppressed. Second, low volatility and low surface activity also contribute to a stable ESI spray, reducing the ESI source contamination. Finally, unlike conventional low molecular mass, MoMs can be used even at high molar concentrations for enantioseparation and tolerate a high concentration of organic solvents in the buffer.

## 4. Development of Chiral Selectors for CE-MS

The search for a novel CS that is also MS-compatible for CE-MS is an emerging topic amongst researchers due to the continuous demand for new chiral separations and sensitive detection methods. Because new chiral molecules are introduced in pharmaceuticals, food, and agricultural industries, so new CS is needed. In the past decade, the exploitations of different selectors for various CE-MS modes were reported in 41 articles by several research groups. The distribution of all these articles based on various modes of chiral CE-MS is shown in Figure 1, whereas Table 1, Table 2 and Table 3 provide separation and detection conditions. The following sub-sections will discuss the utilization of these CS for each chiral CE-MS mode.

### 4.1. Chiral Selectors in EKC-MS and LE-EKC-MS

The moving selectors for EKC, MEKC, and LEKC should meet specific requirements, such as solubility in a volatile buffer, and be stable enough to sustain ionization compatible with electrospray and no interference with the MS signal. The CS used in EKC-MS are low molecular weight, neutral, or charged with multiple chiral centers but are generally incompatible with ESI-MS. Therefore, when EKC is coupled to MS, three approaches are performed using (a) PFT, (b) CMT, and the full-filling technique (FFT). The first two approaches are discussed in Section 3.4, whereas FFT allows both the analyte and CS to enter the MS detector. However, in FFT, the concentration of CS must be very low to allow adequate MS sensitivity, which can sometimes compromise the enantioselectivity. During this review period, 12, 6, and 9 articles reported using PFT, CMT, and FFT, respectively, for EKC-MS. 

The list of CS published between 2011 and 2020 that deals with EKC-MS application in the separation and determination of enantiomeric compounds on different sample types are summarized in Table 1. For EKC-MS, 26 articles are reported. This percentage distribution includes (native and derivatized CD derivatives (30%, 8 articles)), charged CDs (sulfated, highly sulfated, and carboxylated (38.5%, 10 articles)), crown ethers (15.4%, 4 articles), macrocyclic antibiotics (vancomycin 7.7%, 2 articles), dual selectors, DS (7.7%, 2 articles) and 1 article using FLEC as chiral derivatizing reagent. The only CS for LEKC-MS used is a cationic imidazolium β-CD combined with copper sulfate (row 28, Table 1).

**Table 1:** Different types of chiral selectors and their applications in EKC-MS and LEKC-MS.

Chiral Selector	Conditions and Sample Preparation	Application	Ref.
Alpha cyclodextrin (*α*-CD) 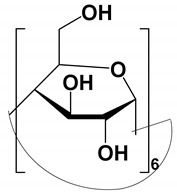	Column: Polybrene coated capillary (50 µm id; 360 µm od; and 60 cm effective length) BGE: 40 mM NH_4_OAC. Injection and voltage: 50 mbar, 5s-24 kV. PFT: 70 mM *α*-CD: SL: MeOH/H_2_O/-NH_4_OH (50/50/−0.2, *v*/*v*/*v*), 10 µL/min ESI-QTOF-MS: Negative −3.5 kV; fragmentor voltage, 135 V; skimmer voltage, 65 V; acq rate: 1.03 spectra/s, DGF: 5 L/min; DGT: 150 °C; NP: 5 psi. SLE: JA standard at 250 ng/g fresh weight in MeOH/-H_2_O (80/20, *v*/*v*) for extraction, overnight agitation at 4 °C, centrifugation at 16,000 rpm for 15 min at 4 °C. Supernatant collected, evaporated to remove MeOH, aqueous residue adjusted to pH 2.8 with acetic acid. LLE: the acidic residue extracted with an equal volume of diethyl ether, discarding the aqueous phase, the organic fraction was vacuum evaporated. Before analysis, the solid residue was resuspended in MeOH/H_2_O (50/50, *v*/*v*) and filtered through a 0.22-µm filter.	Four JA: (i)(3*S*,7*S*)(+)-JA, (3*R*,7*S*)(+)-epi-JA, (3*R*,7*R*)(-)-JA, (3*S*,7*R*)(-)-epi-JA stereoisomers in wounded tobacco leaves.	[28]
Beta cyclodextrin (β-CD) 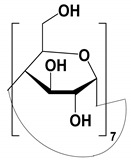	Column: BFS capillary (50 µm id; 360 µm od; 80 cm effective length) BGE: 50 mM NH_4_HCO_3_, pH 8, H_2_O/IPA (85/15 *v*/*v*), 10 mM β-CD. Injection and voltage: 0.5 psi, 20 s, +25 kV. FFT: 10 mM β-CD SL: MeOH/H_2_O/1M NH_4_HCO_3_ (50/50/1, *v*/*v*/*v*), 3 µL/min. ESI-IT-MS: −2.5 kV; fragmentor voltage, 135 V; skimmer voltage, 65 V; acquisition and scan rate: 350 Hz, 110–650 *m*/*z*, DGF: 4 L/min; DGT: 325 °C; NP: 2 psi LLE: 250 mL of thawed CSF sample mixed with 1500 mL of ice-cold (−20 °C) ACN vortex, wait 5 min, centrifuged 15 min, 4 °C 1500 rcf	13 FMOC AAs spiked in human CSF	[29]
Beta cyclodextrin (β-CD)	Column: BFS capillary (50 µm id; 360 µm od; 75 cm effective length) BGE: 49 mM NH,OAc, pH 9.2 15% *v*/*v* IPA, FFT 6 mM β−CD. Injection and voltage: 50 mbar, 5 s, +25 kV SL: 50:50 *v*/*v* IPA, 10 mM NH,OAc, 6 μL/min SQ-ESI-MS: +3.5 kV; fragmentor voltage, 135 V; skimmer voltage, 65 V; acquisition and scan rate: 350 Hz, 110–650 *m*/*z*, DGF: 4 L/min; DGT: 300 °C; NP: 3 psi Preconcentration: LVSS-PS 50 mbar, 60 s, electroosmotic voltage −25 kV, 40 s; separation voltage +25 kV. LLE: 200 µL of thawed CSF mixed with 800 µL of ice-cold (−20 °C) ACN vortex vigorously, wait 5 min, centrifuged 10 min, 4 °C 1200 rcf. The supernatant was collected, dried, reconstituted in 200 µL of 200 mM Na_2_B_4_O_7_ (pH 9.2) Derivatization: A 200 µL 3-hydroxyapatite in 200 mM Na_2_B_4_O_7_, pH 9.2, or the CSF reconstituted sample mixed with 200 µL of 18 mM FMOC-Cl in ACN, vortexed for 8 min, extracted with 0.5 mL n-pentane to remove excess FMOC-Cl and the aqueous phase diluted 1:1 with water before injection	4 stereoisomers of FMOC derivatized 3-hydroxy aspartic acid spiked in rat CSF	[30]
Gamma cyclodextrin (γ-CD) 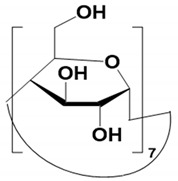	Column: BFS capillary (50 µm id; 360 µm od; 100 cm effective length) BGE: 50 mM ammonium carbonate (pH 10.0), 0.75 mM γ-CD. Injection and voltage: 50 mbar, 15 s, +30 kV. SL: isopropanol/25 mM ammonium carbonate (50/50 *v*/*v*), 3.3 μL/min. FFT: = 0.75 mM γ-CD ESI-IT-MS: +4.5 kV; *m*/*z* range scanned from 100 to 600 with a scan resolution corresponding to 13,000 per second. CID with fragmentation amplitude of 1.0 V; DGF: 3 L/min; DGT: 350 °C; NP: 2 psi. D and F: 100 µL of AAs standards or fertilizer mixture solutions (enzymatically hydrolyzed), diluted 0.1 M borate, pH 10.0, reacted with 200 µL (15 mM)FITC in acetone, kept in the dark for 16 h before injection.	14 AAs in 4 hydrolyzed protein fertilizers	[31]
Gamma cyclodextrin (γ-CD)	Column: BFS capillary (50 µm id; 360 µm od; 60 cm effective length) BGE: 100 mM ammonium formate adjusted to pH 2.6, 20 mM γ-CD. Injection and voltage: 5 kPa, 9 s, +30 kV. FFT: 20 mM γ-CD SL: MeOH/water/formic acid (50/49/1, *v*/*v*), 4 μL/min ESI-QTOF-MS: +3.5 kV; fragmentor, skimmer, and Oct 1 RF voltage of 200 V, 65 V, 600 V, respectively. The *m*/*z* scan range of 150–3000, acquisition rate: 5 spectra/-sec, DGF: 4 L/min; DGT: 275 °C; NP: 15 psi	Standard solution of daclatasvir and its enantiomer	[32]
2-Hydroxypropyl-β-cyclodextrin (HP-β-CD) 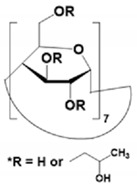	Column: BFS capillary (50 µm id; 360 µm od; 100 cm effective length) BGE: 20 mM ammonium formate, 10% (*v*/*v*) formic acid Injection and voltage: 0.5 psi, 12 s, +24 kV PFT: inject in a plug of 10% (*v*/*v*) formic acid 20 mM HP-β-CD (10 psi, for 1.68 min) SL: 0.1% (*v*/*v*) formic acid, MeOH/water (80:20, *v*/*v*), 2 µL/min using flow microvial interface without sheath gas and nebulizer pressure ESI-QQQ-MS: +3.0 kV; a declustering potential of 98 V, CE: 35 Ev. The *m*/*z* scan range of 150–3000, acq rate 5 spectra/-sec. DGF: 10 L/min LLE: Corydalis Rhizoma extracted using 70% (*v*/*v*) ethanol	*D*/*L* tetrahydro-palmatine and *R*/*S* tetrahydrober-berine in Corydalis Rhizoma extract	[33]
2-Hydroxypropyl-β-cyclodextrin (HP-β-CD).	Column: BFS capillary (50 µm id; 360 µm od; 100 cm effective length) BGE: 50 mM ammonium acetate (pH 3.5) PFT: 0.5% *w*/*v* HP-β-CD (930 mbar, 60 s) Injection and voltage: +15 kV, 30 s, +15 kV; field enhanced injection with micelle-to-solvent stacking. SL: 1% acetic acid in 50% MeOH/water, 3 µL/min. ESI-IT-MS: +4.5 kV; The *m*/*z* scan range of 150–3000 and an acquisition rate of 5 spectra/-sec. DGF: 5 L/min; DGT: 300 °C; NP: 3 psi.	A standard racemic mixture of chlorophenir-amine enantiomers	[34]
2-hydroxypropyl-β-cyclodextrin (HP-β−CD)	Column: BFS capillary (50 µm id; 360 µm od; 104 cm effective length) BGE: 150 mM formic acid acetate (pH 3.0), PFT: 0,5% HP-β-CD, 930 mbar, 60 s) Injection and voltage: 50 mbar, 5 s, +30 kV; SL: 0.1% formic acid in 80% MeOH/H_2_O, 3.3 µL/min. ESI-IT-MS: +4.5 kV; The *m*/*z* scan range of 100–400 and with an acquisition rate of 32,000 *m*/*z*/s) 5 L/min; DGT: 200 °C; NP: 3 psi. SLE: Five capsules homogenized, the ground powder dissolved in DMSO, centrifuge for 10 min at 4000 rpm (20 °C), bring to a known volume and diluted with triply deionized water as needed	Standard mixture of *R*/*S* duloxetine. Quantitation of *S*- duloxetine in pharmaceutical formulation	[35]
Sulfated beta-cyclodextrin (S-β-CD) 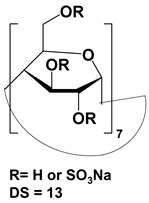	Column: BFS capillary (50 µm id; 360 µm od; 40 cm effective length). Connecting column: (50 um i.d., 5 cm, 30 μm tapered) BGE: 25 mM ammonium phosphate (pH 2.3) CMT: using 0.1% S-β-CD Injection and voltage: 10 cm, 5 s; −300 V/cm and +300 V/cm for separation and connection capillary SL: MeOH/water/formic acid (50/50/0.1 (*v*/*v*/*v*), 3.3 µL/min ESI-IT-MS: Positive, +4.5 kV SLE: Aqueous methyldopa tablets spike with a standard solution of methyldopa	DL-MDOPA (2-amino-2-methyl-3-(3,4-dihydroxy)-phenyl)propanoic acid) in methyldopa tablet	[21]
Sulfated beta-cyclodextrin (S-β-CD)	Column: Bare fused silica capillary (25 µm id; 365 µm od; 65 cm effective length). BGE: 40 mM sodium borate buffer pH 9.5, with FFT: 60 mg/mL S-β-CD Injection and voltage: 100 mbar, 5 s; +30 kV SL: a 20-times diluted buffer was introduced by self aspiration to the nebulizer inlet, where it was mixed with an effluent from the CE capillary. ICP-MS: RF power of 1550 W, plasma gas flow rate of 14.95 L/min, auxiliary gas-flow rate of 0.9 L/min, nebulizer gas-flow rate of 1.08 L/min, make-up gas-flow rate of 0.2 L/min, and a dwell time of 200 ms for ^194^ Pt and ^195^ Pt isotopes. C and F Urine centrifuged for 10 min at 10,000 rpm, spiked with oxaliplatin 0.1 mg/mL solution, and filtered using 0.45 µm PTFE syringe filters.	2 oxaliplatin enantiomers ((*R*,*R*)- Oxaliplatin), and ((*S*,*S*)- Oxaliplatin) in spiked human urine samples	[24]
Sulfated beta-cyclodextrin (S-β-CD)	Column: BFS capillary (75 µm id; 360 µm od; 80 cm effective length) BGE: 200 mM formic acid PFT: 5.0 mM S-β-CD Injection and voltage: 50 mbar, 12 s, +30 kV. SL: 50% MeOH in water containing 0.1% formic acid, 3.0 µL/min. ESI-IT-MS: +4.0 kV; sheath gas 20 psi, au gas 0 psi C and F: Cells cultured in RPMI with 10% heat-inactivated horse serum and 5% fetal bovine serum. 500 μM DOPA (racemic or enantiomer) incubated with 6 mL of PC-12 suspension (2 × 10^6^ cells/mL), 2 h at 37 °C. Cells removed by centrifugation, deproteinized with 300 μL of 30% trichloroacetic acid. Filtered and diluted 10×	DOPA and tyrosine and its precursors in PC-12 nerve cells	[36]
Sulfated beta-cyclodextrin (S-β-CD)	Column: BFS capillary (50 µm id; 360 µm od; 75 cm effective length) BGE: 20 mM acetic acid/-ammonium acetate, pH 5.5 PFT: using 1.0 mM S-β-CD Injection and voltage: 100 mbar, 50 s, +25 kV. SL: 50% MeOH in water containing 0.1% acetic acid, 2.0 µL/min. ESI-IT-MS: +4.0 kV; DGT: 220 °C; NP: 20 psi. C and F: Epinine dissolved in 50 mM phosphate buffer, pH 7.4, mixed with acetaldehyde to final concentrations of 10 and 30 mM for epinine and acetaldehyde, respectively, incubated at 37 °C, 2 h, centrifuged at 10,000× *g* (10 min). Supernatant collected for quantitation	1,2,3,4-tetrahydroisoquinoline derivatives (*R*/*S*)-salsolinol, *N*-methyl salsolinol in incubation solution	[37]
Sulfated beta-cyclodextrin (S−β-CD)	Microchip channel, 4 cm long × 60 µm wide × 20 µm deep; glass substrate, PDMS cover tapered into thin layers (<200 µm in thickness) served as the nano ESI emitter, fixed on an XYZ-translational stage, positioned nanoESI emitter tip ~1.0 mm away from MS orifice. BGE: 10 mM ammonium acetate/acetic acid buffer (pH 5.5) and MeOH (1:1); running buffer at 100 nL/min; PFT: 35 nL 15 mM sulfated β-CD solution at 100 nL/min. Injection and voltage: 600 V, 15 s. ESI-IT-MS ion source voltage, +1.5 kV; relative collision energy 25% with an isolation width of 2.0 u and 30 ms activation time; auxiliary gas, 0 unit, sample capillary temperature, 250 °C. D and F: Cells harvested and suspended in PBS at a 2 × 10^6^ cells/mL density. A portion of the cell suspension (25 µL) was transferred to the sample reservoir, racemic DOPA was added at a final concentration of 100 µM.	DOPA, glutamine, and serine in living cells.	[38]
Sulfated beta-cyclodextrin (S-β-CD)	Microchip channel, 4 cm long × 60 µm wide × 20 µm deep; glass substrate PDMS tapered into thin layers (<200 µm thickness) as the nano-ESI emitter fixed on an XYZ-translational stage, nano-ESI emitter tip ~1.0 mm away from the MS orifice PFT: 35 nL sulfated β-CD solution infused to partially fill the separation channel by turning on and off the syringe pump set at 200 nL/min BGE: 15 mM ammonium acetate/acetic acid buffer (pH 5.5) and MeOH (1:1); running buffer at 100 nL/min; 15 mM sulfated-β-CD Injection and voltage: 350–1500 V, 20 s. ESI-IT-MS ion source voltage, 0 V; RCE 25% with an isolation width of 2.0 u and 30 ms activation time; auxiliary gas, 0 unit, sample capillary temperature, 250 °C. D and F: PC-12 cells cultured in complete RPMI medium with 10% heat-inactivated horse serum and 5% FBS. Cells preloaded and incubated with D/L serine for 120 min, washed, and suspended in PBS solution before being transferred to the sample reservoir	Intracellular and extracellular levels of *D*-serine and *L*-serine.	[39]
Carboxyethyl-β-cyclodextrin 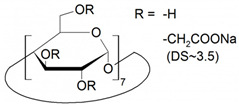	Column: BFS silica capillary (300 µm id; 160 mm effective length) was coated with 0.05% m-hydroxyethylcellulose (*w*/*v*). Preconcentration: An ITP column = 800 μm id poly- tetrafluorethylene capillary of a 90 mm total length BGE: 5 mg/mL of CE-β-CD dissolved in 25 mM ε-aminocaproic acid +25 mM HOAc (pH 4.5) as FFT. Injection and voltage: 50 mbar, 250 s; + 30 kV SL interface: methanol:water (50:50 *v*/*v*), 0.1% (*v*/*v*) acetic acid. 2.0 μL/min. ESI-QqQ-MS: 4.5 kV; DGT: 300 °C; NP: 15 psi; DGF: 5 L/min. Scan range of 100–210 *m*/*z*. D and F: Urine sample frozen immediately after sampling until use. The sample thawed, and each sample was 100-fold diluted with demineralized water and immediately injected	Enantiomers of *N*-(3-phenyl-3-(2-pyridyl)propyl)-*N,N*-Dimethyl-amine maleate in urine	[40]
Highly sulfated gamma cyclodextrin (S-γ-CD) 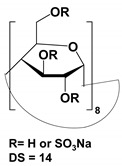	Column: BFS capillary (50 µm id; 360 µm od; 80 cm effective length) BGE: 50 mM pH 2.5 phosphate buffer PFT: 0.6% *v*/*v* HS-γ-CD Injection and voltage 25 kV, 5 kV for 10 s SL: 50:50 *v*/*v* TDI water and MeOH, 5 μL/min ESI-TOF-MS: ESI capillary +3.0 kV, fragmentor 125 V; DGF: 5 L/min, DGT: 180 °C; NP: 10 psi. skimmer: 40 V. D and F: Stock solutions of synthetic cathinones (0.1 mg/mL) in MeOH, filtered and stored at −20 °C.	Separation of 12 cathinones in seized drugs	[41]
Highly sulfated gamma cyclodextrin (S-γ-CD)	Column: Chemically modified sulfonated capillary (50 µm id; 360 µm od; 100 cm effective length) BGE: 10-mM formic acid containing 6% (20 mM) FFT: 20 mM S-γ-CD Injection and voltage: 50 mbar, 5 s, −15 kV. SL: 10-mM NH_4_OAC/-MeOH (50:50, *v*/*v*) 3 μL/min ESI-IT-MS: +4.5 kV; DGT: 200 °C; NP: 5 au, ion-transfer voltage: 550 V D and F. Amphetamine type samples were dissolved in pure water into 50 μg/mL. All sample solutions filtered through 0.25-µm filter	8 amphetamine type stimulants	[42]
Highly sulfated gamma cyclodextrin (S-γ-CD)	Column: BFS capillary (50 µm id;360 µm od; 90 cm effective length). BGE: 50-mM ammonium formate, pH 2.2 FFT: 0.26% highly S-γ−CD. Injection and voltage: 7.25 bar, 10 s, −20 kV. SL: 10-mM ammonium acetate, MeOH/H_2_O (50:50, *v*/*v*), 3 μL/min. ESI-TOF-MS: +4.5 kV; DGT: 180 °C; NP: 4.5 bar, ion-transfer voltage: 550 V D and F. Amphetamines dissolved in water into 0.2 mg/mL (containing 200-µg/mL diazepam as IS), filtered through 0.25-µm filter	20 seized amphetamine and ephedrines-type substances	[43]
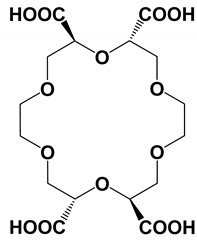 (+)-(18-Crown-6)-2,3,11,12 tetra-carboxylic acid (18C_6_H_4_)	Column: BFS capillary (15 µm id; 360 µm od; 100 cm effective length). BGE: 30 mM 18C_6_H_4_ CMT 30 mM 18C_6_H_4_ in water Injection and voltage 1 psi, 4 s, +25 kV, 5 psi. Sheathless interface: porous capillary tip ESI-OT-MS: +1.1 kV; Mass spectra recorded in the range of *m*/*z* 550–650 *m*/*z* using a scan speed of >4 Hz	Standard racemic mixtures of cathinone, pregabalin, and norephedrine in bath salts	[25]
(+)-(18-Crown-6)-2,3,11,12 tetra-carboxylic acid (18C_6_H_4_)	Column: Polybrene coated fused silica capillary (5–10 µm id; 360 µm od; 100 cm effective length). BGE: 30 mM 18C_6_H_4_ CMT with water as BGE Injection and voltage: −4 kV, 2–3 s, −25 kV Sheathless interface: porous capillary tip ESI-IT-MS: ESI capillary (+0.9–1.0 kV); Mass spectra were recorded in the range of *m*/*z* 550–650 *m*/*z* using a scan speed of >4 Hz	Racemic mixtures of cathinone, pregabalin, and norephedrine	[25]
(+)-(18-Crown-6)-2,3,11,12 tetra-carboxylic acid (18C_6_H_4_)	Column: BFS capillary (15–20 µm id; 150 µm od; 95–115 cm effective length). BGE: 30 mM 18C_6_H_4_ in water in CMT mode. Injection and voltage 25–30 kV were applied to the CE inlet electrode with 3–5 psi of forward pressure, and 1.1–1.25 kV was applied to the CE outlet/ESI electrode, pressure (0.1–1 psi) or −2 to −4 kV (2–5 s); running voltage, +25–30 kV Sheathless interface: porous capillary tip ESI-IT-MS: 1.1 kV; Mass spectra were recorded in the range of *m*/*z* 525–700	D/L ratios of aspartic acid in silk textiles.	[44]
(+)-(18-Crown-6)-2,3,11,12 tetra-carboxylic acid (18C_6_H_4_)	Column: BFS capillary (15–20 µm id; 150 µm od; 100–110 cm effective length). BGE: 30 mM 18C_6_H_4_ CMT: 30 mM 18C_6_H_4_ in water Injection and voltage: pressure (0.1–1 psi) or −2 to −4 kV (2–5 s); +25–30 kV Sheathless interface: porous capillary tip ESI-IT-MS: (+1.1 kV); Mass spectra for aspartic acid/18-C-6-TCA complexes (*m*/*z* = 574) SLE: Bone pieces washed with water and methanol. Nearly 1–5 mg of the bone digested in 6 N HCI at 110 °C for 2 h, centrifuged at 14,000 rpm, supernatants removed, dried to complete dryness, and resuspended in 0.1 N HCI (amino acid solution) for CE-MS analysis	D/L ratio of aspartic acid in human archeological bone specimens	[45]
(+)-(18-Crown-6)-2,3,11,12 tetra-carboxylic acid (18C_6_H_4_)	Column: BFS capillary (50 µm id; 360 µm od; 100 cm effective length) BGE: 30 mM 18C_6_H_4_ with water as BGE; PFT: 30 mM 18C_6_H_4_. 50 mbar,17 min to fill approximately 70% capillary Injection and voltage: +24 kV, 50 mbar for 10 s SL: MeOH:water (1:1, *v*/*v*), 5 μL/min with reference mass solutions of 0.15 μM Hexakis(1H, 1H, 3Htetra-fluoropropoxy)phosphazine and 0.075 μM purine added into the sheath liquid solution ESI-QTOF-MS: +4.0 kV, nozzle voltage: 2 kV; DGF: 12 L/min, DGT: 200 °C; mass spectra recorded in the range of 20–1700 *m*/*z*, scan rate of 1.5 spectra/scan. C and F: grain vinegar, black vinegar, and balsamic vinegar analyze. A 500 μL of each sample was filtered through a 3 kDa cutoff filter by centrifugation at 14,000× *g* for 10 min at 4 °C, filtered aliquot diluted with water. To evaluate signal suppression, 20 μL of a DL-AA mixture (50 μg/mL) spiked into vinegar extracts before injection	20 underivatized DL-AAs in 3 different types of vinegar sample	[46]
Vancomycin 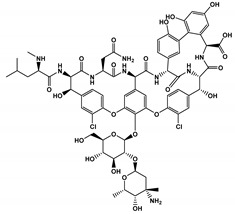	Column: A hexamethrine dibromide coated capillary. (50 µm id; 365 µm od; 100 cm effective length). BGE: 50 mM ammonium formate (pH 7.0), 10 mM vancomycin, 10% MeOH. PFT: partial filling, 150 s × 50 mbar of 10 mM vancomycin in BGE Injection and voltage 50 mbar,150 s, −20 kV. SL interface: 50 mM ammonium formate pH 7.0 (50:50, *v*/*v*) MeOH-water-ammonium hydroxide, 6 μL/min. ESI-IT-MS: −3.5 kV and +4.5 kV; DGT: 200 °C; NP: 6 psi; DGF = 5 L/min. Scan range of 70–440 *m*/*z*.	17 FMOC AAs	[47]
Vancomycin	Column: A polyacrylamide coated capillary (50 µm id; 365 µm od; 60 cm effective length) BGE: 50 mM ammonium acetate (pH 4.5), CMT: 25 mM vancomycin Injection and voltage 100 mbar, 15 s; −25 kV SL interface: MeOH-water-ammonium hydroxide (50:49.5:0.5% *v*/*v*/*v*); 6 μL/min *ESI-QqQ-MS*: −2.5 kV; DGT: 200 °C; NP: 10 psi; DGF = 10 L/min. Mass spectra recorded in SIM (*m*/*z* = 147.1) and MRM (*m*/*z* = 147→129) D and F: The urine sample was diluted 10-fold in triply deionized water, vortexed for 1 min, and filtered using a 0.22 μm syringe filter	D/L-2hydroxyglutaric acid in urine	[48]
Dual chiral selector: 80 mM methyl-β-CD and 40 mM of 2-hydroxypropyl-β-CD	Column: BFS capillary (50 µm id; 365 µm od; 120 cm effective length) BGE: 80 mM methyl-β-CD and 40 mM of 2-hydroxy-propyl-β-CD dissolved in 2 M formic acid (pH 1.2). PFT: 120 cm and 2.5 min (i.e., 83% of the capillary) filled with CDs mixture Injection and voltage: 50 mbar, 250 s; +30 kV SL interface: 50:50 (*v*/*v*) MeOH/water with 0.1% (*v*/*v*) formic acid, 3.3 μL/min ESI-IT-MS: −4.5 kV; DGT: 200 °C; NP: 3 psi; DGF = 5 L/min. Scan range of 100–210 *m*/*z* LLE: Rat plasma samples treated with plasma: acetonitrile, 1:2), centrifuged at 10,000× *g* (15 min, 4 °C), supernatant diluted with formic acid (1:1) to a final concentration of 0.25 M containing trigonelline as IS Filtered with 0.2 μm polytetrafluoroethylene before CE-MS	5 chiral compounds of phenylalanine-tyrosine metabolic pathway: norepinephrine, epinephrine,3,4-dihy-droxyphenylalanine, phenylalanine, and tyrosine in rat plasma	[49]
Dual chiral selector (Crown ether + γ-CD)	Column: BFS capillary (20 µm id; 150 µm od; 95–115 cm effective length). BGE: 0.125%HS-y-CD in 15 mM (+)-18-C-6-TCA PFT: 55% of the capillary is filled with 0.125% HS-y-CD in 15 mM 18C_6_H_4_ FFT: 0.125% HS-y-CD in 15 mM 18C_6_H_4_ Injection and voltage (0.5 or 1 psi) for 4 s, 25 kV Sheathless interface: porous capillary tip. ESI-IT-MS: +1 kV); Mass range of *m*/*z* 560–700 was used to detect the analyte/(+)-18C_6_H_4_ complexes	Positional and optical isomers of amphetamines and cathinones in seized drugs	[50]
6-mono-deoxy-6(4(2aminoethhyl) imidazolyl)−β-CD (CDmh) + Cu(II) complex 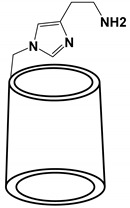	Column: bare fused silica capillary (100 µm id; 365 µm od; 100 cm effective length). BGE: 0.25 mM CDmh CS is dissolved in 2 mM HOAc (pH 4.8), CuSO4 Cu^2+^ and CS1: 1,2 ratio in FFT mode. Injection and voltage: 1 psi, 20 s; +24 kV Preconcentration: LVSS-PS injections of 50 mbar for the 60 s. After injection, a −25 kV for 40 s was applied to electroosmotically pump the sample matrix out of the capillary at the injection end, SL interface: 25:75 *v*/*v* MeOH and 10 mM NHOAc (10 mM), 5 μL/min. ESI-SQ-MS: +4.0 kV; DGT: 250 °C; NP: 3 psi; DGF: 0 L/min. Scan range of 70–1600 *m*/*z*.	Enantiomers of tryptophanate	[51]
(+)-1-(9-fluorenyl)ethyl chloroformate (+) FLEC) as derivatizing reagent 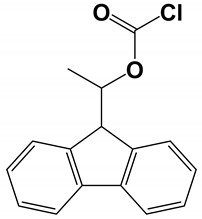	Column: Bare fused silica capillary (50 µm id; 375 µm od; 50 cm effective length). BGE: 150 mM acetic acid, adjusted to pH 3.7 with NH_4_OH. Injection and voltage: 50 mbar, 20 s; −30 kV applied voltage. SL interface: 50:50:0.1 *v*/*v* MeOH:H_2_O NH_4_OH (10 mM); 3 μL/min. ESI-IT-MS: −4.0 kV; DGT: 3000 °C; NP: 6 psi; DGF = 4 L/min; octopole radio frequency amplitude: 115.6 Vp p, capillary exit: −91.0 V, skimmer: −28.4 V, max accumulation time: 50 ms, ion charge control (ICC) target: 200 ms, scan interval: SPE: FLEC derivatives were extracted from the CSF sample using a SPE sorbent, conditioned with 1 mL of MeOH, 1 mL of water. After loading the sample, the cartridge was sequentially washed using 1 mL of aqueous MeOH (15%, *v*/*v*) and 1 mL of water. The FLEC derivatives were eluted by 2 × 0.25 mL of MeOH (containing 0.1% NH_4_OH), eluent was evaporated, reconstituted in 40 µL of ACN: H_2_O (1:1, *v*/*v*)	5 amino acids in artificial CSF	[52]

**BFS:** bare fused silica; **JA:** jasmonic acid; **SL:** sheath liquid; **au:** auxiliary; **PFT:** partial filling technique; **DOPA:** 3,4-dihydroxy phenyl alanine; **FFT:** full filling technique; **BGE:** background electrolyte; **C and F:** centrifugation and filtration; **SL:** sheath liquid; **SPE:** solid phase extraction; **DGT:** drying gas temperature; **DGF:** drying gas flow rate; **D and F**: dilution and filtration; **NP:** nebulizer pressure; **SLE:** solid liquid extraction; **LLE:** liquid-liquid extraction; **ESI-QTOF-MS**: electrospray ionization time of flight mass spectrometry; **ESI-SQ-MS:** electrospray ionization single quadrupole mass spectrometry; **ESI-QqQ-MS:** electrospray ionization triple quadrupole mass spectrometry; **α-CD:** alpha cyclodextrin; **Na_2_B_4_O_7_:** disodium tetraborate; **MeOH**: methanol: **LVSS-PS:** large volume sample stacking polarity switching; **ESI-IT-MS**: electrospray ionization ion trap mass spectrometry; **NH_4_OAC:** ammonium acetate; **NH_4_OH:** ammonium hydroxide; **CMT:** counter-migration technique; **ICP-MS:** inductively coupled plasma mass spectrometry; and FMOC: **FLEC**: (-)-1-(9 -fluorenyl) ethyl chloroformate (FLEC; **CSF:** cerebrospinal fluid).

#### 4.1.1. Native, Derivatized and Charged Cyclodextrins

The CDs are the most utilized and popular sugar-based cyclic macromolecule. The low price and perfect cavity size of native CDs have gained much attention from researchers as this has allowed them to use economic CS for EKC-MS. Native (*α*-, β-, and γ-CDs) were used as neutral chiral selectors in five reports [28,29,30,31,32]. The enantioseparation of four negatively-charged jasmonic acid enantiomers was obtained by PFT using a plug (13.6% of the total capillary length) of 70 mM *α*-CD as a neutral chiral selector in 12 min with LOD about 24 ng/mL [28]. The PFT combined with the CE-QTOF-MS method for jasmonic acids is advantageous to GC-MS as the latter requires a derivatization step. A low concentration of β-CD as CS in EKC-MS was used in two studies [29,30]. Hernández and co-workers used FFT with 10 mM β-CD for the resolution (0.5–1.2) of 15 out of 20 fluorenylmethoxycarbonyl (FMOC) *D*/*L* amino acids (AAs) [29]. The *D*-form of all FMOC-AAs (except for proline) migrates faster than the *L*-form, which should be advantageous in analyzing biological samples where D-AAs are minor components compared to its *L*-antipode. The baseline enantioseparation of four stereoisomers of non-proteogenic AAs such as 3-hyroxyaspartate with two chiral centers was achieved by Liu and coworkers using only 6 mM β-CD in a separate report [30]. The derivatization reagent (FMOC)-chloride (Cl) was found to assist in enantioselective separation. For example, the underivatized 3-hydroxyapatite provided no chiral separation with a BGE of 6 mM β-CD, 49 mM NH_4_OAc (pH 9.2), and 15% *v*/*v* IPA. However, after derivatization, the enantiomers of FMOC-hydroxyaspartate were baseline separated. Furthermore, using a derivatization reagent greatly reduces the amount of β-CD used in enantioseparation, decreases the migration time, and improves ESI-MS detection sensitivity.

Low concentrations of γ-CD as CS in EKC-MS were employed in two separate studies [31,32]. In the first work, 14 chiral AAs were derivatized with fluorescein isothiocyanate (FITC) and enantioseparated with resolutions above 1.0 using β-or γ-CD [31]. However, 0.75 mM γ-CD afforded the more efficient separation of AAs derivatives having two bonded FITC groups than AAs with only one FITC group. Nevertheless, even 5 mM β- or γ-CD was sufficient for the enantioseparation of 11AAs, while higher concentrations, as expected, decreased the signal-to-noise ratio for MS detection. In the second work, the complexation between DCV enantiomers in the presence of γ-CD was studied [32]. Two peaks for DCV with a plateau in between were observed. Experiments were conducted in EKC-MS mode to evaluate the cause of this unexpected phenomenon. NMR and EKC-MS demonstrated the simultaneous presence of 1:1 and 2:1 complexes formed between either DCV, RSSR-DCV, mono-DCV, and γ-CD. Unfortunately, the EKC-MS data showed that both complexes exhibited the same mobility and were not separated, irrespective of the presence or absence of γ-CD.

In this review period, only one derivatized CD, such as 2-hydroxypropyl-β-cyclodex-trin (HP-β-CD), was evaluated as a chiral selector in EKC-MS in three separate reports using the PFT approach [33,34,35]. Yan et al. showed the simultaneous enantioselective analysis of dl-tetrahydropalmatine and (*RS*)-tetrahydroberberine (canadine) in Corydalis Rhizoma extract using the flow-through micro vial CE-MS interface [33]. Although the structures of tetrahydropalmatine and canadine are similar, the concentration of HP-β-CD affected their chiral separations differently. For example, concentrations of 10, 20, 30, 50, and 100 mM of HP-β-CD in the BGE were compared, and the lower concentrations of HP-β-CD resulted in better chiral separation. Canadine migrated slower than tetrahydropalmatine when HP-β-CD concentration was increased. The interaction was too strong to differentiate (*RS*)-canadine when HP-β-CD concentration was 50 or 100 mM. Consequently, 20 mM HP-β-CD ensured simultaneous baseline separation of the two pairs of enantiomers. To our knowledge, this is the first reported study in which chiral EKC-MS is combined with a micro vial CE-MS interface. Two significant advantages of this CE-MS interface are a relatively low flow rate of chemical modifier (1–3 μL/min), and no requirement for the sheath gas. The second advantage essentially eliminates the suction effect that adversely affects the PFT.

Wuetiiric et al. conducted an online sample concentration study in EKC-MS to improve the ESI-MS detectability of chlorpheniramine enantiomers [34]. When using HP-β-CD in combination with PFT and micelle to solvent stacking (MSS), a 500-fold enhancement in LOD was observed. Initially, a long 90 s injection at 30 μg/mL and 15 kV resulted in two poorly resolved and broad peaks. However, using 10 mM ammonium dodecyl sulfate before chlorpheniramine injection sharpened the two enantiomer peaks, indicating the focusing effect of MS on the loaded sample. The 90 s injection provided both enantiomers with a *S/N* of 472, and a calculated LOD of 5 ng/mL (*S/N* = 3). López et al. reported separating a single enantiomeric pharmaceutical drug, duloxetine, using HP-β-CD in PFT mode by chiral EKC-MS [35]. The developed method allows the unequivocal identification of duloxetine enantiomers and improves the sensitivity compared to EKC-UV. For example, the LOD of 200 ng/mL by CE-UV and 20 ng/mL by CE-MS allowed 0.02% of duloxetine enantiomeric impurity profiling. This is the lowest LOD value reported for this drug, satisfying the ICH guidelines requirements.

One of the features of charged CD is the enantioselectivity can be tailored by optimizing the charge interaction and substitution patterns. The self-mobility of charged CDs allows the enantioseparation of both uncharged and charged enantiomers. Additionally, charged CDs improve enantioselectivity for charged analytes due to strong ionic interaction between the oppositely charged species. Although positively-charged CDs are not reported for EKC-MS, negatively-charged sulfated and carboxylated β-CDs and highly sulfated γ-CDs were reported as CS in several reports [21,24,36,37,38,39,40,41,42,43].

The anionic site of these CS offers an electrostatic-supported interaction with cationic guests, enhancing the enantiorecognition capacity at any pHs. Furthermore, this simplifies the EKC-MS method development and helps predict and understand the enantioseparation process since the separation selectivity could be determined as a function of the selector concentration and the pH of the BCE. Several EKC-MS studies compared the enantiorecognition and complexation behavior of the randomly sulfated β- or γ-CD derivatives as well as highly sulfated β- or γ-CD. A significant advantage of sulfated β-CD is that it migrates in a direction away from the MS detector because of its negative charge. A combination of low concentration of only 5 mM sulfated β-CD ensured appropriate chiral selectivity to simultaneously separate six stereoisomers of phenylalanine, tyrosine, and 3,4-dihydroxyphenylalanine (DOPA) in 12 min. The PFT confirmed that this CS improves the MS sensitivity of *L*-DOPA in PC-12 nerve cells [36]. Wu et al. [37] performed in vitro studies using randomly substituted sulfated β-CD. The authors found that *N*-methylsalsolinol (NMSal), a well-known neurotoxin, was formed from the incubation of epinine (a dopamine metabolite) with acetaldehyde (a metabolite of alcohol). The four isomers of NMSal were separated and detected in the incubation solution using 1 mM solution of randomly substituted sulfated β-CD by PFT. The chiral EKC-MS/MS provided the evidence that NMSal exists in four isomeric forms, identified as (*R*)-e.e-NMSal, (*R*)-e.a-NMSal, (*S*)-e.e-NMSal, and (*S*)-e.a-NMSal. Hung and coworkers prepared a desodiated sulfated β-CD by ion-exchange method with the motivation to remove the formation of sodium adduct, increasing ESI-MS sensitivity [21]. Two approaches were investigated. In the first approach, the CMT using a very low concentration (0.1% S-β-CD) and acidic pH allowed the CS to have a net velocity towards the inlet reservoir instead of the ESI source, avoiding the CS entrance in the ESI chamber. The double junction interface allowed the use of nonvolatile phosphate buffer and a continuous supply of S-β-CD to the separation column. In the second approach [21], a carrier mode allowed a very high 2% S-β-CD concentration to be transported towards the junction reservoir. This negatively-charged CD remained in the junction while positively-charged enantiomers of dihydroxyphenylalanine (DOPA) and methyl dihydroxyphenyl-alanine were enantioseparated in 20 min and detected by the ESI source.

Liu’s research group reported fast enantioseparation when 15 mM sulfated β-CD in microchip EKC-MS [38]. Enantiomeric separation of DOPA, glutamic acid, and serine was achieved within 130 s with resolution values of 2.4, 1.1, and 1.0, respectively. The proposed chiral EKC-MS assay provided detection limits of 43 nM and 47 nM for *L*-DOPA and *D*-DOPA, respectively. The microchip EKC-MS provided a single platform for studies on stereochemical preference in living cells because it integrated cell culture, sample injection, chiral separation, and MS detection. In a second communication [39], the research group of Liu reported the same type and concentration of sulfated β-CD to separate enantiomers of *D*/*L* serine in PC-12 cells. After incubating the cells with racemic serine, both intra- and extracellular levels of *D*-Ser and *L*-Ser were quantitated by chiral microchip EKC-MS. Šebestová et al. optimized the enantioseparation of an anticancer metallodrug, oxaliplatin, by EKC-ICP-MS using sulfated β-CD [24]. The concentration of 60 mg/mL was found to be the best because the higher concentration of the CS led to instabilities in the electric current due to Joule heating. Carboxyethyl-β-cyclodextrin (DS 3.5) was an effective CS to separate pheniramine and its metabolite using a combination of EKC and ITP [40]. Piešanskẏ et al. used this CD in 2015 to obtain LOD as low as 80 ng/mL for the above-mentioned chiral compounds in urine.

The negatively-charged highly sulfated (HS)-γ-CD was favorable CS for the enantioseparation of neutral and cationic compounds in EKC-MS. The largest possible enantioselectivities of 12 cathinone analogs were achieved at low pH by varying the concentration of HS-γ-CDs [41]. At concentrations lower than 0.6% HS-γ-CD, some cathinone enantiomers were unresolved, whereas concentrations higher than 0.6% provided excessive noise. Even though a PFT approach was used, the authors suspected that HS-γ-CD entered the ESI-MS source. Therefore, 0.6% HS-γ-CD was the optimum concentration to test the analysis of cathinone in bath salt samples. Mikuma and coworkers used a 10 mM formic acid running buffer at pH 2.5 to prepare 20 mM HS-γ-CD [42]. A chemically modified sulfonated capillary provides repeatable EOF to enantioseparate eight amphetamine-type stimulants. Excellent repeatability of migration with RSD < 0.3% allowed identification of these drugs based on migration times and mass spectra in seized drug samples. In the work of Cui et al., EKC coupled to micro TOF-MS was used for the simultaneous enantioseparation of seven amphetamine-type stimulants (ATS) along with ephedrine using 0.26% HS-γ-CD in 40 min [43]. Interestingly, increasing the HS-γ-CD concentration from 0.20 to 1.06%, the ESI-MS sensitivity of many ATS was not affected despite prolonged separation time. A reverse CE polarity and positive ESI-MS were used so that ATS could be dissociated from the CD complex to enter the MS detector. Notably, the researcher reported no loss of MS sensitivity after six months of usage.

#### 4.1.2. Crown Ethers

Crown ethers are macrocyclic polyethers that can form selective complexes with alkali, alkaline earth, and primary amines. Crown ethers first introduced by Kuhn and coworkers for chiral separation of 20 amines in EKC in 1992 [53]. Based on the chiral separation data, two recognition mechanisms were proposed. The first mechanism assumes that the four ether substituents act as a barrier for chiral molecules. In the second mechanism, electrostatic interactions occur between negatively-charged carboxylate group on crown ether (host) anions and positively-charged amines (guest) molecules. Thermodynamic data validated the mechanism. Two observations were noted. The best chiral resolution was achieved if the chiral center of the targeted compound was adjacent to amine functionality. However, an excellent enantioresolution was obtained for compounds with a chiral center relative to the amine group in the delta position.

Several studies listed in Table 1 (rows 19–23) compared the enantiorecognition and complexation behavior of one derivative of crown ether as CS by adding (+)-(18- crown-6)-2,3,11,12-tetracarboxylic acid ((+)-18-C_6_H_4_-TCA, MW 440) to the CE-MS background electrolyte derivatives. Moini’s group presented two examples of using (+)-18-C_6_H_4_-TCA as CS to measure the AAs racemization rate [44,45]. In the first example, a new sensitive and fast EKC-MS method was developed using a 30–60 mM solution of 18-C-6-TCA in water as the BGE and complexation reagent [44]. Under this condition, 11 *D*- and *L*-AAs were separated and detected as protonated amino acid/18-C-6-TCA complexes in a single run. For example, aspartic acid was detected at *m*/*z* 574 (to increase the detection sensitivity in the attomole range. The method was developed for dating silk textiles by measuring the *D*/*L* ratio of the aspartic acid for several well-dated textiles ranging from the present to ~2500 years ago. In a related report [45], the same research group studied the racemization rate of aspartic acid in human bone and other mammals. The studies suggested that the racemization rate is species-specific.

In a third study on the use of (+)-18-C_6_H_4_-TCA published by Moini’s group, ultrafast EKC-MS was combined with polybrene coated capillary to achieve baseline separation of underivatized optical isomers of amphetamine, cathinone, normephedrone, and pregabalin with an analysis time shorter than two minutes [25]. Chiral resolutions of 1.3, 3.7, and 3.8 were achieved for pregabalin, cathinone, and normephedrone, respectively, using only 30 mM (+)-18-C_6_H_4_-TCA conditions in formic acid buffer and a CMT approach. Lee and coworkers used variable concentration (i.e., 30–50 mM (+)-18-C_6_H_4_-TCA) with and without PFT to separate 17 AAs [46]. Without PFT, the underivatized, AAs under acidic conditions formed complexes with (+)-18-C_6_H_4_-TCA showing ions of (M+(+)-18C_6_H_4_-TCA +H)^+^ in a positive ESI spectrum. Ions of the free AA ions, (M+H)^+^, protonated 18C_6_H_4,_ and its salt adduct was also identified. The relative ratios depended on the amount of CS used. Except for proline and asparagine, chiral resolution values ranged from 0.5 to 21.0. These results included baseline separations of 11 AAs. The *L*-AAs migrated faster than the *D*-AAs except for serine, threonine, and methionine. To reduce contamination of the ESI source by (+)-18-C_6_H_4_-TCA, the PFT approach followed the reduction of the separation zone length to 70% of the capillary, filled with 30 mM 18C_6_H_4_ in water. This method showed higher sensitivity but no resolution of several AAs than without PFT. The separated AA peaks were observed as free AA ions, (AA+H)^+^ with detection limits (LODs) ranging from 0.07 to 1.03 µg/mL.

#### 4.1.3. Vancomycin

Vancomycin (VC) is a charged and non-volatile CS (Table 1, row 24–25) that causes ion suppression in ESI-MS. To date, only one type of macrocyclic antibiotic, vancomycin (VC), is investigated for chiral EKC-MS. Hernández and coworkers studied the potential of VC as CS for the EKC-MS/MS of AAs for the first time in 2014 [47]. A total of 17 AAs were derivatized with FMOC-Cl to improve their interaction and, consequently, chiral separation with VC. A non-covalent cationic coating using hexadimethrine bromide and a PFT approach allowed increased MS^2^ sensitivity. The EKC-MS/MS approach enabled enantioseparation in 20 min with LOD in the range of 0.2–3.1 μM. The chloride salt of 25 mM VC was used as a CS for the enantioseparation of 2-hydroxyglutaric acid enantiomers in a capillary full-filling method in EKC-MS/MS [48]. A resolution of nearly 2 with a total separation time of 9 min allowed quantitation from 0.13 to 400 µmol/L of 2-hydroxyglutaric acid with satisfying accuracy and precision.

#### 4.1.4. Dual Chiral Selectors

There are several excellent reported examples in the literature for EKC-MS when using a single CS is insufficient to obtain efficient chiral separation of the enantiomers [10]. Therefore, enantioresolution was improved using a dual CS system in those cases. The EKC with tandem mass spectrometry was reported using a dual CS system containing methyl-β-CD and 2-HP-β-CD in a molar ratio of 2:1 for the simultaneous enantioseparation of five chiral compounds of the phenylalanine-tyrosine metabolic pathway (norepinephrine, epinephrine, 3,4-dihydroxyphenylalanine, phenylalanine, and tyrosine) metabolites using a 2 M formic acid at an extremely acid pH of 1.2 [49]. Different combinations of native and derivatized CDs were tested. Interestingly, methyl-β-CD is the CD that most influences the resolution of each chiral analyte, whereas HP-β-CD affects the simultaneous enantioseparation of the analytes of interest. Thus, using a dual CD system consisting of a mixture of 100 mM methyl-β-CD and 60 mM HP-β-CD, in which 83% of the capillary filled with the CDs offers synergistic effects. Moini and coworkers use a combination of 0.125% HS-γ-CD and 15 mM (+)-18-C_6_H_4_-TCA utilizing a low rate (~10 nL/min) to achieve enhanced resolution of positional and optical isomers of six seized drugs: (±)-amphetamine; (±)-methamphetamine; positional and optical isomers of 3-fluoromethcathinone (3-FMC); 4-fluoromethcathinone (4-FMC); (±)-methylone; and (±)-pentylone [50]. As expected, the FFT always provided higher resolution compared to PFT. The enhancement in resolution observed was due to (+)-18-C-6-TCA in the BGE.

#### 4.1.5. Miscellaneous

There is only one reported study falling under scenario ligand exchange (LE)-EKC-MS, using 6-mono-deoxy-6(4(2aminoethhyl) imidazolyl)-β-CD (CD-mH) derivative as CS. The analyte complex was used to separate tryptophan racemate by LE principles [51]. The addition of a ligand (central metal ion (i.e., copper (II)) to the CS (i.e., CD-mH)) improved the chiral selectivity by introducing an additional steric parameter that affected the binding properties of the CD to the analyte. The two diastereomeric complexes of copper(II)-CD-mH and tryptophan were detected separately (i.e., different ionic currents at the same concentration), pointing toward the importance of generating separate calibration curves. For example, it is noted that an *m*/*z* = 758 due to the presence of [Cu(CDmh)-(TrpO)^+^] Na^+^ was also detected, and this is attributed to the presence of doubly charged complex in combination with the sodium ion. The authors emphasized the higher sensitivity of ESI-MS over ultraviolet detection, and we concur that the LE-EKC-MS has potential for non-UV active chiral compounds analysis.

Finally, a different approach to chiral separation in EKC-MS is to chemically convert chiral analytes to their respective diastereoisomers by derivatization with a chiral derivatization reagent to allow stable diastereomers to be separated under achiral conditions. The apparent advantage of this indirect method is the possibility of reducing the LOD of the diastereomers by MS detection. However, the range of chiral compounds that this approach can separate is mainly limited to their functional groups. The reaction times for each analyte-derivatizing reagent combination can vary extensively (i.e., from several minutes to 24 h), still, the excess reagent and its by-products are formed. A chiral derivatizing reagent, such as (±)-1-(9-fluorenyl)ethyl chloroformate (FLEC), has shown to have very useful for the analysis of five relevant *DL*-AAs after derivatization with (+)-FLEC [52]. The separation of *DL*-serine, *DL*-asparagine, *DL*-aspartic acid, *DL*-glutamine, and *DL*-glutamic acid was achieved in 22 min. The resolution of AA diastereomers depended on the volatile BGE (acetic acid titrated with ammonium hydroxide) pH. The elution order reversal was observed for aspartic acid derivatives. The optimized pH for achieving the separation of all AAs in one run was 3.7.

### 4.2. Chiral Selectors in MEKC-MS

In chiral MEKC-MS, chiral recognition is based on the partitioning of the chiral analyte between a MS-compatible micellar phase and the surrounding volatile BGE. Thus, micelles are formed using a covalently stabilized polymeric surfactant (also known as MoMs) with zero CMC or volatile surfactant monomers that form micelles in a volatile aqueous solution above their CMC. The first documented study that used MoMs for chiral MEKC-MS was performed by Shamsi in 2002 [8], who showed that polysodium *N*-undecenyl-*L*-valinate (poly-*L*-SUV) could be used to separate racemic binaphthyl mixture. In the past decade, several MS-compatible polymeric surfactants have been used. The most frequently used surfactants suitable for chiral MEKC-MS are AA and peptide-based polymeric surfactant [54,55,56], sugar-based polymeric surfactants [57], mixtures of polymeric surfactants [23], and achiral volatile surfactants, such as ammonium perfluorooctanoate (APFO) [58,59]. APFO is an achiral, but MS-compatible, volatile surfactant used in a system involving indirect chiral separation employing chiral derivatizing reagents (Table 2)*. A variety of MEKC-MS surfactants listed in Table 2 offer possibilities for improved enantioseparations for a broad range of analytes without the need for a PFT approach.

**Table 2:** Different types of chiral selectors and their applications in MEKC-MS.

Chiral Selector	MEK-MS Conditions	Application	Ref
Polysodium *N*-undecenoyl*-L,L*-leucylvalinate (poly-L,L-SULV) 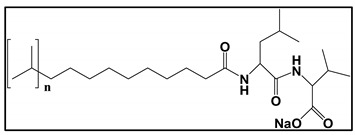	Column: Bare fused silica capillary column (118 cm L, 50 um i.d., 375 um o.d) BGE: 25 mM NH_4_OAc pH 5.0 FFT:25 mM poly-L,L-SULV with 15% MeOH. Injection and voltage: 5 mbar for 2 s; 30 kV. SL: MeOH/H_2_O (80/20, *v*/*v*), 5 mM NH_4_OAc at pH 6.8; 5 µL/min ESI-SQ-MS: −3 kV; fragmentor voltage, 91 V; DGF: 6.0 L/min; DGT: 200 °C; and NP: 4 psi. The ESI-MS detection was performed in the SIM mode. [M−H]^−^ ions were monitored at 307 *m*/*z* WAR and 323 *m*/*z* for 4′-, 6-, 7-,8, 9 and 10-OH WAR ESI-QqQ-MS: same as for SQ-MS except for DGF: 8.0 L/min; fragmentor voltage set at 125 V for WAR and OH-WAR and 75 V for I.S.; collision energy at 20 eV for WAR and OH-WAR and 5 eV for I.S. SPE: MAX cartridges pre conditioned with 2 mL of MeOH, 2 mL of water. Aliquots (250 µL) of plasma sample, 2.5 µL of I.S. solution and 250 µL of 10% (*v*/*v*) perchloric acid or 4% (*v*/*v*) phosphoric acid were transferred to a 1.5 mL microcentrifuge tube. After vortex mixing for 30 s, centrifugation (10 min at 1000 rpm, (9279 g)], the supernatant (450 µL) loaded on a preconditioned MAX cartridge. The loaded sample was washed with 2 mL of 2% (*w*/*w*) NH_4_OH, 2 mL of H_2_O, eluted with 2 mL of MeOH	Warfarin and its five hydroxylated metabolites in plasma	[54]
Polysodium *N*-undecenoyl-*L,L*-leucylalaninate (poly-*L,L*-SULA) 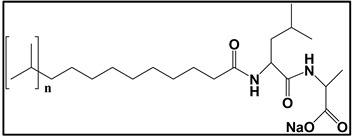	Column: Bare fused silica capillary of 60 cm length, 50 µm i.d. 365 mm o.d. BGE: 20 mM NH_4_OAc, 25 mM TEA, pH 8.5 FFT: 15 mM poly-L,L-SULA Injection and voltage: 5 mbar, 100 s, +25 kV. SL: 5 mM NH_4_OAc in 80/20 MeOH/H_2_O, pH 6.8; 5 µL/min. ESI-QqQ-MS DGF: 8 L/min; NP: 3 psi; DGT: 200 °C; capillary voltage 3000 V; fragmentor 113 V SPE: Strata-X-C polymeric strong cation cartridges (3 cc, 60 mg).activated with 2 mL of MeOH, 2 mL of TDI water. Aliquots of 250 µL patient plasma sample, 2.5 µL of IS (1 mg/mL) and 250 µL of 4% (*v*/*v*) H_3_PO_4_ were added to a 1.5 mL tube. After vortexing for 30 s, the mixed solution was centrifuged at 1000 rpm for 10 min. Supernatant (~450 µL) was loaded on the pretreated Strata-X-C cartridge, allowed to be adsorbed into the cartridge for 15 s, the loaded sample on the cartridge was washed with 2 mL of 0.1 M HCI and 2 mL of MeOH, dried under gentle stream of air, 30 s. The analytes eluted with 2 mL NH_4_OH/-MeOH (5/95 *v*/*v*) in a 10 mL glass tube, solvent was evaporated in a vacuum chamber under a gentle stream of air. The residue in the tube was reconstituted with 50 µL of MeOH/H_2_O (10/90, *v*/*v*) before injecting	Venlafaxine, *O*-desmethylvenlafaxine, *N*-desmethylvenlafaxine in human plasma	[55]
Polysodium *N*-undecenoxycarbonyl-*L*-leucinate (poly- *L*-SUCL) 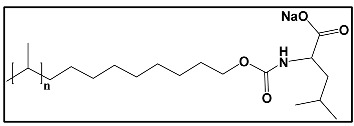	Column: A 77 cm fused silica capillary column covalently bonded with 28 mg/mL of 2-acryl-amido-2-methyl-1-propanesulfonic acid BGE: 20 mM NH_4_OAc, 15 mM TEA FFT: 25 mM poly*-L* SUCL, pH 8.8 Injection and voltage: 10 psi, 20 s; +30 kV SL interface: 25:75 *v*/*v* MeOH and 10 mM NH_4_OAc (10 mM). 5 μL/min ESI-QqQ-MS: 3.5 kV; DGT: 200 °C; NP: 3 psi; DGF = 6 L/min SLE: Two tablets of meto and two tablets of aten, total weight of 313.6 mg and 180.0 mg, respectively, crushed, transferred separately to two separate 100 mL flask, add 50–60 mL of MeOH, sonicated for 30 min for complete dissolution, diluted up to the mark with MeOH and shaken well, filtered separately. A diluent contains 400 µL of meto, 400 µL of aten solution,125 µL of the pindo (IS), 75 µL of MeOH, 9 mL of TDI-H_2_O, providing a final concentration of 20 mg/mL of aten and meto	(+/−)Atenolol and (+/−) metoprolol in commercial tablets using pindolol as IS. standard, a racemic mixture of norphenyl-ephrine, ephedrine and atropine	[56]
Dual polymeric chiral surfactant Polysodium *N*-undecenoyl*-L,L*-leucyl-valinate (poly-*L*-SULV) + polysodium *N*-undecenoxycarbonyl-*L*-leucinate (poly-*L*-SUCL)	Column: A bare fused silica capillary of 120 cm length, 50 μm i.d. 365 mm o.d. BGE: 25 mM NH_4_OAc, pH 8.0, with 75 mM poly-SULV and 25 mM poly-SUCL FFT: 75 mM poly-L-SULV + 25 mM poly-L-SUCL Injection and voltage: 5 mbar, 10 s, +25 kV. SL: 5 mM NH_4_OAc in 50/50 MeOH/H_2_O, 0.5% *v*/*v* acetone; flow rate 7.5 μL/min APPI-SQ-MS DGF: 5 L/min; NP: 5 psi; DGT: 150 °C; vaporizer temperature 150 °C; capillary voltage 2000 V; fragmentor 80, gain 3; SIM at *m*/*z* = 195, 197	A standard mixture of 4 benzoin derivatives	[23]
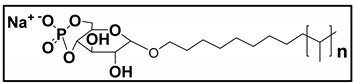 Polysodium N-undecylenyl-alpha-D-glucopyranoside 4,6-hydrogen phosphate (poly-*α* -*D*-SUGP) Polysodium *N*-undecylenyl-alpha-D-glucopyranoside 6-hydrogen sulfate (Poly-*α*-D-SUGS) 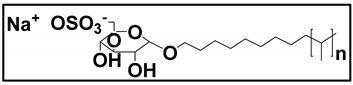	Column: Bare fused silica capillary of 60 cm length, 50 μm i.d. 365 μm o.d. BGE: 20 mM NH_4_OAc, pH 10.8 FFT: 15 mM (poly-*α*-*D*-SUGP with 15 mM (poly-*α*-*D*-SUGP or poly-*α*-*D*-SUGS) Injection and voltage: 5 mbar, 10 s, +15 kV. SL: sheath liquid: 5 mM NH_4_OAc in 80/20 MeOH/H_2_O, pH 6.8; 5 μL/min ESI-QqQ-MS +3 kV, DGF: 6 L/min; NP: 3 psi; DGT: 250 °C; fragmentor voltage and collision energy varied for different compounds from 83–200 and 17–41, respectively	Standard mixture of 1,1′-binaphthyl phosphate, ephedrine, pseuodoephedrine, methyl-ephedrine, norephedrine, atenolol, metoprolol, carteolol, talinolol	[57]
Ammonium perflurooctanoate (APFO) 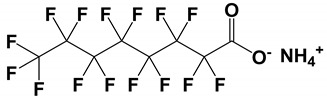	Column: Bare fused silica capillary of 80 cm length, 50 μm i.d. 365 μm o.d. BGE: 150 mM APFO (pH 9.5) FFT: 150 mM APFO Injection and voltage: 0.5 psi, 10 s: SL: isopropanol-water-formic acid; (90:10:0.1, *v*/*v*/*v*), 3 μmL/min ESI-IT-MS: +500 oV, NP: 2 psi, DGT: 300 °C, DGF 4 L/min LLE: The buffer concentration and pH of the CSF samples were adjusted by adding 260 µL of 200 mM sodium tetraborate (pH 9.2) and 10 µL of 2 M sodium hydroxide to 1740 µL CSF. To 180 µL AA standard mixture in 26 mM sodium tetraborate or pH adjusted CSF, 180 µL of 12 mM FLEC solution diluted with acetonitrile) were added and mixed, yielding a FLEC/AA concentration ratio between 50 and 100. The mixture was incubated for 10 min at room temperature. The sample was 1:1 diluted with water to enhance analyte stacking	(+ )-1-(9-fluorenyl)ethylchloroformate derivitized *DL*-AAs spiked in human CSF	[58]
Ammonium perflurooctanoate (APFO)	Column: Bare fused silica capillary of 60 cm length, 50 mm i.d. 365 mm o.d BGE: 150 mM APFO (pH 9.5); FFT: 150 mM APFO Injection and voltage: 0.5 psi, 10 s SL: sheath liquid, isopropanol-water-formic acid (90:10:0.1 *v*/*v*/*v*); 2.5 µL/min ESI-IT-MS: +5000 V, NP: 138 mbar, DGT: 300 °C DGF: 4 L/min; octopole RF amplitude: 168.9 Vpp, capillary exit: 48 V, skimmer: 29.7 V, Oct 1 DC: 11.7 V, Oct 2 DC: 1.8 V, max accumulation time: 300 ms, scan interval: 200–650 *m*/*z* LLE: Two aqueous artificial CSF solutions (500 mL each) were separately prepared in water. (A) NaCl (8.66 g), KCI (0.224 g), CaCl_2_-2H_2_O (0.206 g) and MgCl-6H_2_O (0.163 g); (B) Na_2_HPO_4_-7H_2_O (0.214 g) and NaH_2_PO_4_-H_2_O (0.027 g). The final artificial CSF solution prepared by mixing A and B. The samples prepared by spiking six AAs acids in artificial CSF, followed by a 1:1 dilution with 5 mM sodium tetraborate and 10% (*v*/*v*) ACN	(+)-1-(9-fluorenyl)ethyl chloroformate derivitized 6 AAs in artificial cerebrospinal fluid	[59]

**FFT:** full filling technique; **BGE:** background electrolyte; **SL:** sheath liquid; **SLE:** solid-liquid extraction; **DGT:** drying gas temperature; **DGF:** drying gas flow rate; **NP:** nebulizer pressure; **LLE:** liquid-liquid extraction; **SPE:** solid-phase extraction; **ESI-QTOF-MS**: electrospray ionization-time of flight mass spectrometry; **ESI-SQ-MS:** electrospray ionization single quadrupole mass spectrometry; **ESI-QqQ-MS:** electrospray ionization triple quadrupole mass spectrometry; **APPI-SQ-MS:** atmospheric pressure photoionization ionization single quadrupole mass spectrometry; **ESI-IT-MS**: electrospray ionization ion trap mass spectrometry; **NH_4_OAC:** ammonium acetate; **NH_4_OH:** ammonium hydroxide; **CMT:** counter-migration technique; **TDI:** triply deionized: **CSF**: cerebrospinal fluid; and **MeOH:** methanol.

#### 4.2.1. Direct Chiral Separations in MEKC-MS

Much of the past 10 years of research for direct chiral separation in MEKC-MS has been focused on amino acid or dipeptide-based polymers derived from undecylenic acid, with carbamate and amide linkages functionalized with single amino acid or dipeptide head group [23,54,55,56]. Using 25 mM poly-sodium *N*-undecenoyl-*L,L*-leucylvalinate (poly *L,L*-SULV) as a chiral pseudophase in MEKC-MS baseline enantioseparation of warfarin, its five metabolites along with the internal standard (coumachlor) was obtained in 45 min [54]. This separation time was much faster than 100 min required to enantioseparate the same mixture with a packed chiral column via CEC-MS. Another dipeptide polymeric surfactant such as poly-sodium *N*-undecenoyl-*L,L*-leucylalaninate (poly-*L,L*-SULA) simultaneously enantioseparated venlafaxine (*VX*), and two of its metabolites (*O*- and *N*-desmethyl VX) in 25 min [55]. A baseline enantioseparation of both *O-DVX* and *VX* enantiomers was achieved in 15 min after optimizing the buffer pH, poly-*L,L*-SULA concentration, nebulizer pressure, and separation voltage. The LOD was 30 ng/mL and 21 ng/mL for *O*-DVX and VX, respectively. Jun et al. developed a simultaneous separation by chiral MEKC and online atmospheric pressure photoionization (APPI)-MS detection of four chiral photoinitiators: hydrobenzoin, benzoin, benzoin methyl ether, and benzoin ethyl ether (BEE) [23]. Using an optimized molar ratio of 85:15 and a mixed molecular micelle of two polymeric chiral surfactants (polysodium N-undecenoxy carbonyl-L-leucinate (poly-*L*-SUCL) and polysodium *N*-undecenoyl-*L,L*-leucylvalinate (poly-*L,L*-SULV) all four chiral benzoins mentioned above were enantioseparated in 45 min. The chiral MEKC-MS conditions, such as voltage, mixed polymeric surfactant concentration, buffer pH, and BGE concentration, were optimized using a multivariate central composite design (CCD). Additionally, the sheath liquid composition and spray chamber parameters were also optimized with CCD. Models based on the CCD results and response surface method were constructed to analyze the interactions between MEKC separation and MS detection factors and their effects on the responses. The final optimum conditions for CMEKC-APPI-MS were also predicted and found in agreement with the experimentally optimized parameters.

Although MEKC-MS using bare silica capillary with MoMs is a useful hyphenated technique for chiral analysis, it is still challenging because of the lower repeatability of retention time and peak area associated with the difficulty in controlling EOF on bare silica capillaries. Furthermore, the fused-silica capillary tip lacks robustness, and electrospray erosion limits the capillary column’s lifetime. To overcome these challenges, Akter et al. recently proposed the covalently bonded 2-acrylamido-2-methyl-1-propane-sulfonic acid (AMPS) column filled with polysodium *N*-undecenoxy carbonyl*-L*-leucinate (poly-*L*-SUCL) [56]. Simultaneous enantiomeric separations and MS/MS detection of three β-blockers were achieved within 25 min with an improved column lifetime of at least 45–50 runs. Excellent repeatability of retention time was observed for β-blockers, as evidenced by the relative standard deviation of less than 2% and 3% for intra-capillary and inter-capillary columns, respectively.

Yijin and coworkers introduced a new MS-compatible sugar-based polymeric surfactant for chiral MEKC-MS [57]. Two phosphate surfactants such as polysodium *N*-undecenylenyl-alpha-*D*-glucopyranoside 4,6-hydrogen phosphate (poly-*α*-*D*-SUGP) and polysodium *N*-octenylenyl-alpha-*D*-glucopyranoside 4,6-hydrogen phosphate (poly-*α*-*D*-SOGP), two sulfated sugar surfactants, such as polysodium *N*-undecenynl alpha-*D*-glucopyranoside 6-hydrogen sulfate (poly-*α*-*D*-SUGS) and poly-sodium *N*-octenyl-alpha-*D*-glucopyranoside 6-hydrogen sulfate (poly-*α*-*D*-SOGS), were tested for enantioseparation of two class of cationic drugs (ephedrines and β-blockers). These polymers show more significantly enhanced MS signal intensity than their monomer counterparts with LOD of 10–100 ng/mL.

#### 4.2.2. Indirect Chiral Separations in MEKC-MS

The indirect chiral separation in MEKC-MS is possible when chiral analytes are derivatized with a chiral reagent to form corresponding stable diastereomers separated using an achiral volatile surfactant. The main advantage of the indirect method is reducing the contamination of the ionization source in ESI-MS with a nonvolatile CS, consequently improving the MS detection sensitivity of the optical isomers. Therefore, using a non-volatile chiral surfactant in MEKC is circumvented by employing FLEC as a chiral derivatizing agent and ammonium perfluorooctanoate (APFO) as a volatile achiral pseudostationary phase for separation of the formed diastereomers of AAs without ion suppression in two separate reports [58,59]. In the first study, efficient AA derivatization with FLEC was completed within 10 min [58]. The effect of the pH of the BGE, APFO concentration, capillary temperature, sheath-liquid composition, and flow rate as well as spray chamber settings, were tested to prevent analyte fragmentation and achieve sensitive ESI-MS detection. Selective detection and quantification of 14 proteinogenic AAs were performed with chiral resolution in the range of 1.2–8.6 and LOD from 130 to 630 nM. Moldovan et al. [59] showed that derivatization improves enantioselectivity and MS detection. Although in-capillary derivatization in chiral MEKC brings significant analysis time, this drawback is offset by implementing a continuous workflow. Thus, precapillary derivatization must be fast and quantitative, the reaction by-products should not interfere with the enantioseparation, and all reaction products should be compatible with the MS detection method. A fully automatized MEKC-MS method with in-capillary derivatization was developed for the chiral analysis of 17 *D*- and *L*-AAs using FLEC as a derivatizing reagent. The procedure was optimized using a multivariate experimental design leading to the following conditions: sample and FLEC plugs in a 2:1 ratio (15 s, 30 mbar: 7.5 s, 30 mbar) followed by 15 min of mixing using a voltage of 0.1 kV. Using a BGE consisting of 150 mM APFO as a volatile surfactant at pH 9.5, the diastereomers of 8 out of 14 AAs were entirely separated. At the same time, 6 AAs were partially resolved and detected in the range of 3–11 μM [59].

### 4.3. Chiral Selectors in CEC-MS

The CS immobilized on the capillary wall in a CEC-MS mode has zero mobility to travel towards the detector end. Thus, it results in no contamination of the ionization source of the MS instrument. The immobilized CS makes CEC a perfect tool in chiral separations when hyphenated to MS to yield sensitive detection. Table 3 lists zero, two, and four articles published on open-tubular, packed, and monolithic chiral CEC-MS, respectively, between 2011 and 2020.

**Table 3:** Different types of chiral selectors and their applications in CEC-MS and LE-CEC-MS.

Chiral Selector Approach	CEC-MS Conditions	Application	Ref
Sulfonated cellulose tris (3,5 dimethyl carbamate (CDMPC-SO_3_) 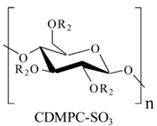	Packed column (75 um i.d.; 363 μm o.d.) cut to the appropriately packed bed lengths (15 cm for the optimized inlet side, 35 cm for the optimized outlet side) coated with 20% CDMPC-SO_3_. Mobile phase: 70%ACN, 5 mM NH_4_COOH, pH 3.5, Injection and voltage: injection at 6 kV, 6 s, +25 kV runs. SL: sheath liquid, MeOH/-H_2_O (90:10, *v*/*v*), 50 mM NH_4_OAc, flow rate, 5.0 µL/min ESI-SQ-MS: capillary voltage: +3000 V; fragmentor voltage: 80 V; DGF: 5 L/min; DGT:130 °C; NP: 4 psi. The SIM mode was set at *m*/*z* = 233 to monitor the desired [M+H]^+^	(+/−)amino- glutethimide	[15]
GMA-β-CD-2-acrylamido-2-methyl-propane sulfonic acid (AMPS) 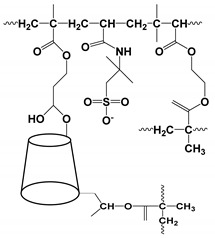	Monolithic column Capillary (100 um i.d.; 365 um o.d.) were cut to the appropriate total capillary length of 50 cm with a 25 cm portion of the monolith in the inlet CE side, open 25 cm portion of the capillary placed in the MS outlet side. Mobile phase: 50/50 ACN/H20, 5 mM NH_4_OAc, 0.3% *v*/*v* TEA, pH 4.0. Injection and voltage: Injection at 5 kV, 3 s, +30 kV, 12 bar pressure from the inlet side. SL: sheath liquid, MeOH/-H_2_O (80:20, *v*/*v*), 5 mM NH_4_OAc, flow rate, 5.0 µL/min. ESI-SQ-MS: capillary voltage, +2000 V; DGF: 5 L/min; DGT: 200 °C; NP: 5 psi; fragmentor voltage 84 V, DGF: 3 L/min; DGT: 150 °C; NP: 20 psi; SIM positive ion mode.	Standard solution of 6 enantiomers of hexobarbital, catechin, flavanone, trogers base, aminoglutethimide, pseudoephedrine	[18]
Cellulose tris (3,5 dimethyl carbamate 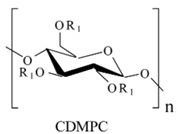 (CDMPC) Sulfonated cellulose tris (3,5 dimethyl carbamate (CDMPC-SO_3_)	Packed column internal taper at the outlet end (50 cm × 100 µm i.d. capillary; 7 cm or 20 cm packed with 5 µm 1000° Å bare silica particles coated with 20% CDMPC and CDMPC-SO_3_ BGE: Variable %ACN, 5 mM NH_4_COOH, pH 3.5, 20 kV, 25 °C Injection and voltage: electrokinetic at 10 kV, 10 s. SL: MeOH/-H_2_O (90:10, *v*/*v*), 50 mM NH_4_OAc, flow rate, 5.0 µL/min ESI-SQ-MS: capillary voltage: +3000 V; fragmentor voltage: 80 V; DGF: 5 L/min; DGT: 130 °C; NP: 4 psi. SIM mode set at corresponding polarity to monitor the protonated [M+H]^+^ or deprotonated [M−H]^−^ molecular ions	Standard solution of (+/−) warfarin, (+/−) glutethimide, (+/−) aminoglutethimide, trifluroanthryl-ethanol	[60]
GMA-β−CD vinyl benzyl trimethylammonium (VBTA) 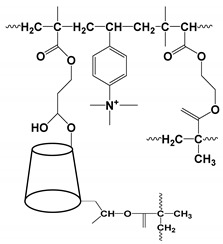	Monolithic column Capillary (100 or 150 um i.d.; 365 um o.d.) were cut to the appropriate total capillary length of 50 cm with a 25 cm portion of the monolith in the inlet CE side, open 25 cm portion of the capillary placed in the MS outlet side Mobile phase: 75/25 ACN/H_2_O, 5 mM NH_4_COOH, pH 3.0 Injection and voltage: 50 mbar 15 s, −30 kV, 2 bar pressure from the inlet side. SL: sheath liquid, MeOH/-H_2_O (50:50, *v*/*v*), 5 mM NH_4_OAc, flow rate, 4.0 µL/min. ESI-QqQ-MS: capillary voltage: −3500 V; DGF: 3 L/min; DGT: 150 °C; NP: 20 psi; fragmentor voltage 84 V, precursor ion of 284 *m*/*z* and product ion of 178 *m*/*z*	Standard solution of (atrolactic acid, 2-(3-chlorophenoxypropionic acid, 2,4-dinitro-phenyl-threonoine)	[61]
Sodium 10-acryl-amidodecenoxy carbonyl-L-leucinate (SAADCL) 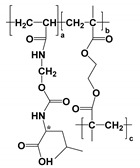	Monolithic column Capillary (100 µm i.d.; 365 um o.d.) cut to the appropriate total capillary length of 53 cm, 30 cm portion of the monolith in the outlet CE side, open 25 cm portion of the capillary placed at MS inlet side. Mobile phase: 5 mM NH_4_OAc, 0.5% (*v*/*v*) TEA (pH 5.0), 70% (*v*/*v*) ACN. Injection and voltage: 5 kV, 3 s, +15 kV, 2 bar pressure SL: sheath liquid, MeOH/-H_2_O (80:20, *v*/*v*), 5 mM NH_4_OAc, flow rate, 8.0 µL/min ESI-QqQ-MS: capillary voltage, +3500 V; DGF: 5 L/min; DGT: 150 °C; NP: 7 psi; MRM product ions observed at *m*/*z* 115.1 and 133.1	Standard solution of ephedrine and pseudo ephedrine	[62]
L-pipecolic acid 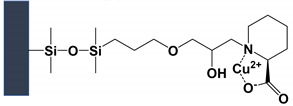	Monolithic column 100 μm i.d.; 365 um o.d. capillary cut to the total capillary length of 70 cm with an effective monolithic bed of 33 cm. Mobile phase: 50 mM ammonium acetate adjusted with acetic acid to pH 6.5 ACN:H_2_O (7:3, *v*/*v*), preinjection: mobile phases in the absence of Cu(II) ions for 12 h postinjection: 16 mM CuSO4, 30 min Injection and voltage: 50 mbar 15–20 s, −10 kV SL: sheath liquid, MeOH/-H_2_O (50:50, *v*/*v*), 5 mM NH_4_OAc, flow rate, 4.0 µL/min. ESI-IT-MS: capillary voltage: +20 V; ion-spray voltage: 3500 V; tube lens voltage 35 V. DGF: 4 L/-min; DGT: 150 °C; NP: 8 psi; scan: *m*/*z* 100–1000, scan time:200 ms.	Standard solution of 4 dansylated (DNS) +/− amino acids (DNS-valine, DNS-phenylalanine, DNS-threonine, DNS-serine)	[63]

**BGE:** background electrolyte; **SL:** sheath liquid; **SLE:** solid-liquid extraction; **LLE:** liquid-liquid extraction; **SPE:** solid-phase extraction; **DGT:** drying gas temperature; **DGF:** drying gas flow rate; **NP:** nebulizer pressure, **ESI-QTOF-MS**: electrospray ionization-time of flight mass spectrometry; **ESI-SQ-MS:** electrospray ionization single quadrupole mass spectrometry; **ESI-QqQ-MS:** electrospray ionization triple quadrupole mass spectrometry; **ESI-IT-MS**: electrospray ionization ion trap mass spectrometry; **NH_4_OAC:** ammonium acetate; and **NH_4_OH:** ammonium hydroxide.

#### 4.3.1. Packed Column

A novel fritless chiral column packed with sulfonated polysaccharide for CEC-MS was reported by Bragg et al. [15] (Table 3, row 1). The column fabrication process involves the formation of internal tapers on two separate columns. Once the internal tapers are formed and the columns are packed, the untapered ends of each column are joined together by a commercially available connector. The central composite design multivariate analysis optimized the best *S/N* and resolution with the shortest possible retention time were used as criteria to optimize the inlet and outlet taper diameters for the chiral separation of (±)-aminoglutethimide. The fritless system shows improved efficiency (due to its reduction of bubbles formation) as there was no fritted material providing better robustness and repeatability of migration times. To meet the growing demand for rapid chiral analysis, high throughput chiral separations were achieved with the combined use of a 7 cm column packed with highly charged CDMPC-SO_3_ CSP in CEC-MS [60]. Significantly shorter separation time with baseline resolution of four chiral compounds (trifluoroanthryl ethanol, warfarin, aminoglutethimide, and glutethimide) were obtained on the 7 cm column mentioned above than the standard 20 cm column length packed with the same CSP. We concur with the author’s conclusion that the concept of using a short chiral CEC column packed with CSP capable of generating strong EOF is ideal for quick screening of a combinatorial library of chiral compounds to find the lead compound in drug discovery.

#### 4.3.2. Monolithic Columns

Another type of fritless column is the use of monolithic stationary phases. There were three new polymeric chiral phases [18,61,62], but only one silica-based chiral monolith [63] was introduced in this review period. Using chiral monomers to make monolithic columns in one step is an attractive approach. The first report on the use of polymer-CD-based monolithic column for chiral CEC-MS was introduced by Gu and Shamsi in 2011 [18]. A novel synthetic monomer, the glycidylmethacrylate-bonded β-CD was synthesized, conveniently copolymerized with commonly used charged monomers (aminopropyl sulfonic acid, AMPS), crosslinker (ethylene dimethacrylate (EDMA)) in the presence of porogens (butanediol, propanol, and dimethylsulfoxide) to form a rigid rod and a versatile chiral cation exchange monolithic column in few hours. The monolithic columns showed enantioseparation of hexobarbital, catechin, pseudoephedrine, and four other analytes as test chiral compounds. Although the ESI parameters were not extensively optimized, CEC-ESI-MS still showed several folds higher *S/N* than CEC-UV for all of the generated chromatograms of each analyte. The column was tested and demonstrated excellent stability over 60 injections for separation of hexobarbital enantiomers over two days with homogenous microflow and no loss of monolithic material at the CEC-MS interface. For example, good interday repeatability with per cent RSD for retention times, selectivity, and resolution of 3.4–4.7%, 1.7%, and 4.2%, respectively, were achieved, suggesting that the GMA-β-CD monolith was tightly anchored to the capillary wall.

In a second report (Table 3, row 4), a vinylbenzyl trimethylammonium (VBTA) as a novel positively-charged achiral co-monomer was copolymerized with the above-mentioned glycidyl methacrylate-bonded β-CD monomer to prepare anion exchange monolithic column with anodic EOF [61]. This anion-exchange monolith column was tested for both chiral CEC-UV and CEC-MS and was found to be beneficial for the analysis of acidic enantiomers. Initial efforts to test this monolithic column for CEC-MS experiments were challenged by the low flow rates and the anodic EOF produced by the GMA-β-CD-VBTA monolithic column. Two modifications to the system were used to counteract this problem. First, it was necessary to switch to a slighter higher ID column from 100 μm to a 150 µm ID to increase the flow rate. Second, it was necessary to turn the segment of the capillary containing monolith towards the inlet CE side of the cassette and the open capillary segment at the MS outlet end of the CE instrument. Hence, this flip of the open and monolithic segments resulted in successful elution and separation of 2-(3-chlorphenoxy) propionic acid, 2,4-dinitrophenylthreonine, and atrolactic acid. When coupled to a QqQ-MS, the same monolithic CEC column provided two orders of magnitude higher sensitivity than a SQ-MS instrument.

Jun and Shamsi [62] showed the potential of a novel AA-bound chiral surfactant monolithic column for CEC-MS (Table 3, row 4). By designing a system with an acryloyl amide tail, carbamate linker, leucine head group of different surfactant chain lengths, and a conventional crosslinker, a new “one-pot” synthesis for the generation of the AA-based polymeric monolith was possible. Three novel chiral surfactant-bound C_8_-C_12_ monolithic columns were tested for the enantioseparation of ephedrine (EP) and pseudoephedrine (PEP) enantiomers. Although the C_12_ carbon chain length monolith provided the best resolution and selectivity of (±)-PEP, the efficiency was lower, and the run times were slightly longer. However, a 10-carbon chain surfactant column was more versatile than 8 and 12 carbon chain surfactant columns in separating a wide range of positively-charged chiral compounds. Nevertheless, the data on CEC-MS/MS for simultaneous separation and detection of (±)-PEP and (±)-EP with a sodium 10-acryl-amidodecenoxy carbonyl-*L*-leucinate (SAADCL) column suggest great potential for using this type of monolithic column configuration for sensitive detection in CEC-MS/MS.

Only one report (Table 3, row 3) utilizes a silica-based chiral monolithic chiral column for CEC-MS. Zhang and co-workers modified pre-formed a silica monolithic column by a sol-gel process following modification with *L*-pipecolic acid as the chiral stationary phase for CEC-MS [63]. The ability of the column to hold copper(II) ions after column preparation and conditioning allowed for chiral ligand exchange separations of racemic dansyl AAs without copper ions in the mobile phase, allowing successful ESI-MS detection.

## 5. Applications

Due to the non-compatibility of CS with ESI-MS, there are not a plethora of applications for CE-MS in the past decade. In addition, many modes of CE-MS, such as CEC-MS, LEMEKC-MS, LECEC-MS, and microchip CE-MS, are still in their developmental stage. In contrast, considerable investigations in EKC-MS and MEKC-MS analyzed different types of chiral compounds from different categories of real-life samples (column 3, Table 1 and Table 2). Stereoselectivity is evaluated in biological specimens (plasma, urine, cerebrospinal fluid (CSF), and bones), pharmaceuticals (prescription drugs, seized drugs), cell cultures, plants and plant extracts, and miscellaneous (textiles and vinegar) studies.

### 5.1. Biospecimen

In the past 10 years, several chiral components were measured in various biospecimens, such as cerebrospinal fluid (CSF), urine, plasma, and bone, and have been enantio-separated by EKC-MS. The related articles published are tabulated with corresponding CE-MS conditions in the last two columns of Table 1 and Table 2 and are further discussed in the following sub-sections.

The CSF is a transparent body fluid present in the brain and spinal cord. The composition of CSF is similar to that of plasma but with a substantially lower content of proteins (less than 1 g/L compared to 70 g/L in plasma) and different composition of salts. Analysis of CSF by CE is advantageous because CSF volumes can be withdrawn are limited. Several research groups developed the analysis of derivatized AAs in rat and human CSF samples as D-AAs are recognized as signaling molecules in the nervous system. Thus, the chiral analysis of AAs is reported in several publications [29,30,52,58,59].

A total of 13 DL-AAs were successfully resolved in aqueous standard spiked in human CSF [29]. The spiked results showed chiral resolution of 12 out of 13 AAs, except for cysteine, which was not detected due to a poor *S/N* ratio. The LOD of most of the AAs were in the range of 1–21 μM, with a linear response of several AAs in the 3–90 μM range. In addition, the %RSD of peak area and migration time were in the range of 4.9–16% and 0.9–16%, respectively. Because the repeatability of peak area and migration time obtained in CSF are very similar to the standard aqueous solution, matrix effects in CSF for AAs analysis were minimal. To verify the presence of endogenous levels of 3-hydroxyaspartate in rat CSF, the separation of a standard mixture of four stereoisomers of 3-hydroxyaspartate was derivatized with FMOC and simultaneously separated and detected in less than 30 min [30]. As expected with the normal EKC-MS method, no 3-hydroxyaspartate was detected in the rat CSF sample due to the relatively low sensitivity. However, when large-volume sample stacking and polarity switching (LVSS-PS) as a preconcentration method was incorporated into an EKC-MS protocol, low *D*-erythro-3-hydroxyaspartate (D-EHA) levels were detected. Triplicate analysis of the rat CSF sample indicated the significance of the signal with estimated endogenous levels of 977 nM, which was within the reported range for *D*-EHA in human CSF [64].

In a third study, a targeted EKC-MS approach was tested for the chiral analysis of five biologically relevant AAs derivatized with FLEC in artificial CSF [52]. The diastereoselectivity is dependent on pH for all analytes in a BGE composed of 150 mM acetic acid, pH 3.7 with NH_4_OH. Moreover, a reversal of the migration order of aspartic acid enantiomers was observed. This reversal of elution order is believed to be caused by intramolecular hydrogen bonding interactions affecting the *pKa* of the second ionizable carboxyl group in the side chain. The applicability of this method was tested using artificial CSF. A solid-phase extraction (SPE) protocol was developed for the selective extraction of the FLEC derivatives of AAs. A complete assessment of the matrix effect and extraction yield was performed. The evaluation indicated that the matrix effect is marginal, and the recoveries range between 46 and 92%. The EKC-MS method offers adequate sensitivity with LOD of at least 1 µM.

**Figure 6:**
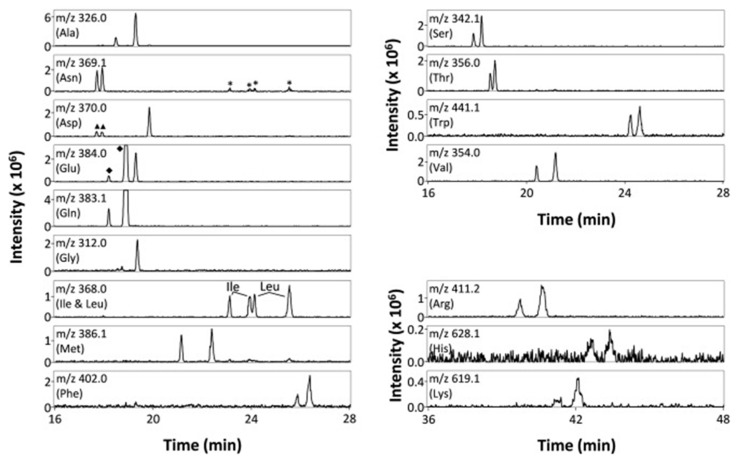
Extracted ion electropherograms obtained during chiral CE-MS of CSF spiked with DL-Ms. Conditions: BGE, 150 mM APFO (pH 9.5); spiked concentration per AA enantiomer, 3.2 µg mL-1 in CSF; sheath liquid, isopropanol-water-formic acid (90:10:0.1 *v*/*v*/*v*); sheath liquid flow rate, 3 µL. **Asterisks** indicate signals of the mono isotope peaks of *D*- and *L*-isoleucine and o- and L-leucine. **Triangles** indicate the mono CB isotope peaks of *D-* and *L*-asparagine. **Diamonds** indicate mono CB isotope peaks of *D*- and *L*-glutamine. Both Figure and Figure caption is adapted from Prior et al. [58], with permission from Elsevier.

The fourth study on CSF [58] was performed by MEKC-MS using a fluorocarbon surfactant to analyze human CSF spiked with *DL*-AAs (Figure 6). Similar to AA standard mixture analysis, 16 DL-AAs and glycine could be detected in one run, from which 14 were enantioseparated with resolutions of 1.3–8.3. However, no enantioseparation was obtained for aspartic and glutamic acid. Note that the peak height observed for the *L*-enantiomer was higher than the peak height of the *D*-enantiomer, suggesting the presence of endogenous *L*-AAS in CSF. The endogenous concentrations of the detected *L*-AAs were estimated based on the spiked concentration and were found to be in the range of 4–71 µM for most *L*-AAs. In a final study [59], the same fluorocarbon surfactant was used for AAs but after in-capillary derivatization with FLEC. The optimized derivatization procedure was tested on artificial CSF spiked with six AAs (alanine, serine, valine, glutamine, methionine, and phenylalanine) diluted two-fold with 5 mM sodium tetraborate at a pH of 9, required for the derivatization. However, unsatisfactory recoveries (compared to the standard samples) were found in the range of 13–19%. The recoveries were significantly improved to 73.7–94.5% by adding 10% acetonitrile (*v*/*v*) to the samples. However, no changes in migration times and resolution values made the authors conclude that the MEKC-MS method is suitable for AAs analysis in CSF.

Urine is a promising diagnostic sample obtained by a non-invasive collection method. Urine testing is considered the gold standard because it has a wide detection window and is less costly as well as less invasive than blood testing. However, there are only three publications for analyzing enantiomers in urine matrix by CE-MS in this review period [24,40,48].

**Figure 7:**
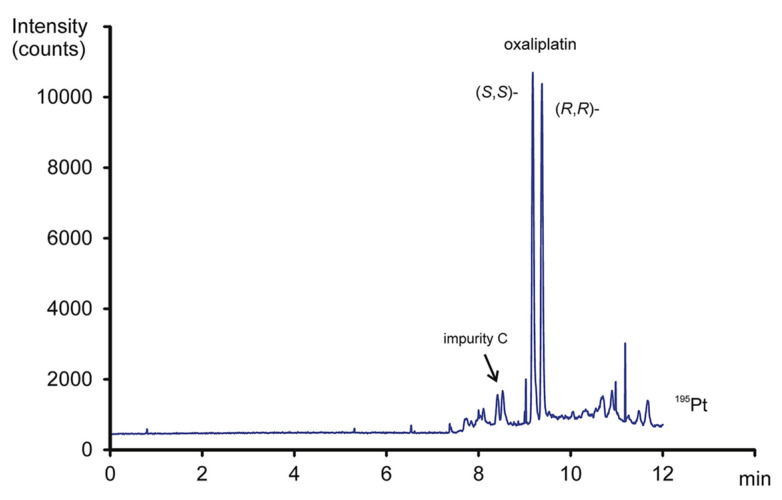
Separation of oxaliplatin enantiomers in urine using CE-ICP-MS. BGE: sodium borate buffer pH 9.5, ionic strength 40 mmolL^−1^, with 60 mg/mL sulfated -β-CD; voltage: 30 kV; monitoring of ^195^Pt isotope. Both Figure and Figure caption is adapted from Sebestova et al. [24], with permission from Elsevier.

Platinum-based cancerostatic drugs, such as oxiplatinin enantiomers, were separated by EKC-MS and detected by ICP-MS [24]. The method was successfully tested in urine samples of healthy volunteers spiked by oxaliplatin enantiomers to the therapeutic level and directly injected into CE-ICP-MS. As shown in Figure 7, both enantiomers of oxaliplatin and impurity C were easily detected.

The platinum-related peaks were, perhaps, due to the hydrolysis of the oxaliplatin in urine. The authors commented that the same samples using CE with UV detection showed too many unspecific signals. No data was published on the concentration effects of a nonvolatile buffers system composed of 40 mM sodium borate and 60 mM sulfated β-CD on the ICP-MS signal. A rather impressive LOD and LOQ values of 64 and 116 ng/mL of oxaliplatin, respectively, indicate EKC hyphenation to ICP-MS as the correct technique to study the chiral metabolism of platinum-based drugs in human urine.

In a second report [40], theraflu was administered orally to six volunteers, and the urine sample was collected at 3, 9, 12, and 24 h to monitor pheniramine and its metabolite. The urine samples (stored −20 °C) were simply thawed and diluted 100-fold before analysis. The chiral ITP-EKC using a large-bore capillary, 5 mg/mL of CE-β-CD, and a QqQ mass spectrometer provided enantioseparation in 45 min with excellent sensitivity. The LOD and LLOQ of 10 and 100 pg/mL were achieved for pheniramine enantiomers.

Low molecular weight carboxylic enantiomers, such as *D*/*L* 2-hydroxyglutaric acid, is a valuable marker for diagnosing inborn error of metabolism. The enantioresolution of 2-hydroxyglutaric acid enantiomers provides a resolution value of 2.05 using VC as a CS [48]. The method was validated and applied to determine 2-hydroxyglutaric enantiomers in urine samples obtained from healthy subjects compared to two urine samples from child patients. The amounts of *D*-isomer were substantially higher than the *L*-isomer of 2-hydroxyglutaric in both child patients compared to healthy controls. The chiral ITP-EKC using a large-bore capillary, 5 μg/mL of CE-β-CD, and a QqQ mass spectrometer provided enantioseparation in 45 min with excellent sensitivity. The LOD and LLOQ of 10 and 100 pg/mL were achieved for pheniramine enantiomers.

Chiral drug concentration is more often measured in plasma rather than whole blood or serum. One possible reason is that blood is composed of plasma and red blood cells (RBCs), adding complexity to the analysis. On the other hand, the serum is the fluid obtained from blood after blood is allowed to clot, and the proteins present in serum are not the same proteins as plasma. Plasma is a liquid tissue segment in which the drug in the plasma fluid equilibrates with the drug in the tissues and cellular components. Measuring the concentration of drug enantiomers in plasma allows us to understand drug metabolism, pharmacological activity, tolerability, safety, and drug–drug interactions.

**Figure 8:**
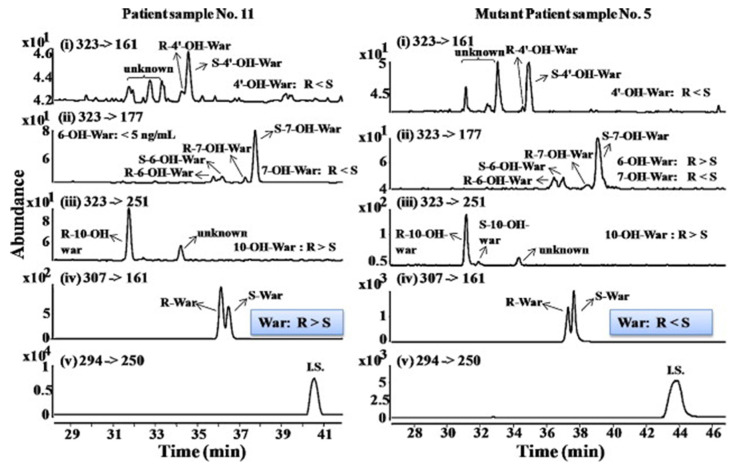
Extracted ion chromatograms of subject 11 and subject 5 (with mutant gene CYP2C9′2 or ′3). The MEKC-MS/MS conditions are the same as in Table 2 (row 2). The figure is adapted from Wang et al. [54], with permission from Elsevier.

Different chiral EKC methodologies have been developed to separate drugs and metabolites in plasma. However, only two works have been reported in the past decade on the simultaneous enantiomeric separation of drugs and their metabolites in plasma by MEKC-MS [54,55]. In the first report, the Shamsi research group compared the *R*/*S* enantiomeric ratio of warfarin enantiomers and their hydroxylated metabolites in normal versus mutant patients’ plasma (Figure 8) [54] using MoMs of sodium *N*-undecenoyl-*L*, *L*-leucylvalinate. As expected, the peak height of *R*-warfarin > S-warfarin in a normal subject (patient 11, left chromatogram) versus mutant subject (patient 5, right chromatogram). In contrast, the trend in the *R*/*S* ratio for hydroxy metabolites in plasma is not reversed. Furthermore, *R*-6-OH-WAR, *R*-10-OH-WAR, *S*-4′-OH-WAR, and *S*-7-OH-WAR were quantified with 85%, 95%, 95%, and 98% recovery in 55 human plasma, respectively. Among 95% and 4% of these human plasma samples, the major observed metabolites were *S*-7-OH-WAR and *R*-10-OH-WAR, confirming the literature report on warfarin metabolism, i.e., the formation of these two major isomers are catalyzed by human CYP2C9 and CYP3A4, respectively.

The second study used the plasma sample to profile drug–drug interactions [55]. Venlafaxine (*VX*) is an antidepressant drug administered as a racemic mixture. However, the two enantiomers of *VX* exhibit different pharmacological activities. Moreover, the conversion of *VX* to *O*-desmethyl-venlafaxine (*O*-*DVX*) and *N*-desmethylvenlafaxine (*N*-*DVX*) is the major and minor biotransformation pathway in humans. Several dipeptide-based MoMs for enantioseparation of *R*/*S* enantiomers of *VX*, *O*-*DVX*, and *N*-*DVX* were achieved. The use of polysodium *N*-undecenoyl-*L, L*-leucylalaninate (poly-L-SULA) was the most enantioselective MoMs for baseline separation of all three isomeric pairs of VX, *O*-*DVX*, and *N*-*DVX* in less than 15 min. Additionally, the LOD and LOQ of 10.5 ng/mL and 31 ng/mL of *VX,* 15 ng/mL and 45 ng/mL of *O*-*DVX,* were obtained. These values were superior to EKC-UV using charged CDs. The chiral MEKC-MS was validated, and the method was also applied to study the potential drug interactions when coadministered with indinavir (a drug used in HIV therapy). The results indicated that in the subject without indinavir therapy, the *S/R* peak height ratio of *O-DVx* enantiomers changes from 0.83 at 1 h to 0.98 at 4 h. On the other hand, a significant decrease in the peak height ratio of *S/R* enantiomers is observed in the subject with indinavir therapy at 4 h compared to 1 h. However, the peak height ratio of *S/R O*-*DVX* is not reversed and remains less than one. Furthermore, the *S/N* ratio of *O*-*DVX* with indinavir therapy is significantly higher than without treatment for both 1 h and 4 h sample collection. These results suggested that the increased concentrations of *R*-(−) and *S*-(+) enantiomers of *VX* and *O*-*DVX* are influenced by indinavir in a similar magnitude, possibly by reduced drug metabolism or excretion from the body.

The plasma specimen was also used in a final study to simultaneously profile all phenylalanine-tyrosine metabolites using a dual CD system composed of 180 mM methylated β-CD and 40 mM 2-hydroxypropyl β-CD in a very acidic formic acid buffer at pH 1.2 [49]. Good chiral selectivity of the developed EKC-MS was achieved, but all analytes had different *m*/*z* values and could be identified by their MS^2^ spectra. The authors commented that one would think that the complete resolution between the individual compound is not necessary as MS can distinguish them. However, the authors noted differences in *m*/*z* values between the metabolites are very small (often only two units of *m*/*z*). Thus, without electrophoretic separation, the isolation of precursor ions is difficult and may cause quantitation errors due to the presence of isotope ions of other metabolites. In addition, coelution may cause ion suppression. Hence, it is desirable to have simultaneous separation of the studied chiral metabolites in several instances, especially when the chemical structure of the compounds is very similar. The superior simultaneous chiral and achiral selectivity with no interfering peaks in the plasma sample resulted in the LOD and LOQ ranging from 40–150 nM to 133–500 nM. The analysis of plasma showed that *L*-Phe and *L*-Tyr were present in the analyzed rat plasma samples at the µM level.

The estimation of *D*/*L* AA helps date human bones. However, the gold standard of human ^14^C dating by radiocarbon method requires a large sample size. The process is expensive, time-consuming, and cannot accurately estimate the dating of bone samples older than 100,000 years. Chiral EKC-MS is nearly 1000-fold more sensitive than ^14^C-dating and consumes a fraction of the sample, even when compared to GC or LC techniques. In addition, EKC-MS overcame almost all of the disadvantages of the dating method mentioned above. Using a bare fused silica capillary and a microliter quantity of a (+)-18-C_6_H_4_-TCA as CS allowed for determining the racemization rate of *D*/*L* aspartic acid and, hence, the process of bone aging [45]. The ratio ranged from 2.4-to 10.0%, with a good correlation between the *D*/*L* ratio and the sample’s age. However, Moini’s group noted that the racemization rate is species-specific, but the developed EKC-MS method can estimate human bones dating less than 10,000 years old.

### 5.2. Pharmaceuticals

This sub-section describes the analysis of two categories of pharmaceuticals (prescription and bulk) drug products. The analysis conditions are listed in Table 1 and Table 2, providing the reader with more detailed information.

A drug that can be obtained only using a physician’s prescription is considered a prescription drug. Four prescription drugs (metoprolol (METOP), atenolol (ATEN), duloxetine (DX), and methyldopa (MDOPA)), in the form of tablets or capsules, were analyzed in three separate reports [35,38,56]. Duloxetine (also known as Cymbalta) is a selective serotonin and norepinephrine reuptake inhibitor. Duloxetine affects chemicals in the brain that may be unbalanced in people with depression. López et al. employed a 0.5% HP-β-CD, 150 mM ammonium formate (pH 3.0) partial filling strategy in EKC-MS to perform the quality control of the drug duloxetine, commercialized as a pure enantiomer [35]. Four different batches of pharmaceutical capsules were analyzed. The values obtained for enantiomeric impurity percentage, duloxetine content, and established range of duloxetine were compared using statistical tests (*F*- and *t*-tests). There were no significant differences between the precision achieved between EKC-UV and EKC-MS methods as measured by the *F*-test, nor between the quantities obtained from the analysis of the drugs (*t*-test). It is worth mentioning that none of the pharmaceutical formulations analyzed by the two chiral EKC-UV and EKC-MS showed any detectable amounts of the enantiomeric impurity and the *S*-duloxetine content was equivalent to the labeled content of 100% value. The prescription drug MDOPA used to treat high blood pressure is a class of antihypertensives that works by relaxing the blood vessels so that blood can flow easily through the body [38]. The EKC-MS with a double junction interface was proposed by Hung et al. to analyze MDOPA in a commercial tablet. Notably, a nonvolatile phosphate buffer with 0.5% (*w*/*v*) of sulfated β-CD was used in this study without significant ion suppression for the MDOPA enantiomers. A peak corresponding to the migration time of *D*-MDOPA was only observed when the tablet solution was spiked with a racemic standard of *DL*-MDOPA.

**Figure 9:**
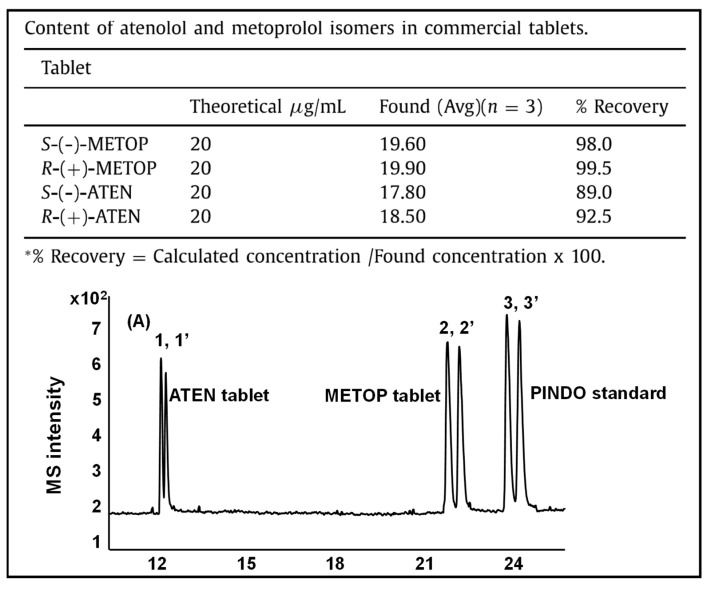
A typical chromatogram for the analysis of a mixture prepared by using: (A) *R.S*-ATEN, and *R,S*-METOP tablets. Peak identification: 1 = *S*-(−)-ATEN, 1’ = *R*-(+)-ATEN, 2 = *S*-(−)-METOP, 2’ = *R*-(+)-METOP, 3 = *S*-(−)-PINDO, 3’ = *R*-(+)-PINDO. All other MEKC-MS conditions are the same as indicated in Table 2 (row 1) except using 5 mbar for 50 s at +30 kV. The inset table shows content of atenolol and metoprolol enantiomers measured in commercial prescription tablets. Adapted from Akter et al. [56], with permission from Elsevier.

A rugged and repeatable MEK-MS method was recently developed using a combination of MoMs, polysodium *N*-undecenoxycarbonyl-L-leucinate (poly-*L*-SUCL), and AMPS bonded column [56]. The accuracy of the MEKC-MS/MS method was tested by measuring the enantiomeric content of commercial ATEN and METO tablets. The electropherogram in Figure 9 and the inset Table represent the analysis of commercial ATEN and METO tablets with pindolol as IS in less than 25 min. Almost 98–99% of the enantiomeric content of the injected METO tablet and 89–92% of the enantiomeric content of ATEN tablets were recovered under optimum conditions. Racemic tablets of ATEN and METO showed a nearly equal portion of (*S*)-(−)- and (*R*)-(+)- for both enantiomers. For example, 51/49 and 48/52 enantiomeric ratios of ATEN and METOP were, respectively, obtained for each beta-blocker during analysis. The LOD and the lower LOQ were 299 ng/mL and 500 ng/mL, respectively, for both beta-blockers.

Seized drugs, including amphetamines, cathinones, or even prescription drugs such as pregabalin, are captured or controlled by law-enforcement agencies. When these drugs are confiscated, law enforcement stores them in a secure warehouse called a crime labora-tory. The drugs are tested, cataloged, organized, and shelved at this location, awaiting criminal trial. In addition, the storage conditions can vary depending on which enforcement agency is investigating. Detecting and identifying controlled substances is crucial in law enforcement’s fight against drug-related crime and violence. Several seized drugs were analyzed in this review period, mainly by chiral EKC-MS. The seized drugs were analyzed in two separate studies by Moini’s group [25,50]. Synthetic cathinones (also known as bath salts) are potent stimulants and psychoactive drug derivatives of the naturally occurring substance cathinone (an amphetamine analog). Cathinone is extracted from the Cathaedulis plant (khat). A sheathless EKC-MS using a 20-µm-I.D. capillary with a porous tip interface separated cathinone derivatives and optical isomers [50]. A combination of 0.125% HS-γ-CD and 15 mM (+)-18-C-6-TCA increased the resolution of cathinone derivatives and their optical isomers, allowing all positional and optical isomers to be baseline resolved in 24 min. In a second study, ultrafast EKC-MS was used to separate three-drug mixtures (*RS*-pregabalin, *DL*-cathinone, and DL-normephedrone) in less than 1.6 min with resolution values of 1.3, 3.7, and 3.8 [25]. A simple polybrene coating with 15 mM (+)-18-C-6-TCA allowed this rapid analysis.

The sulfated γ-CD was utilized in two separate reports to analyze amphetamine-type stimulants and cathinones [41,42]. The EKC-MS has been proposed to analyze seized drugs, including 12 cathinone analogs [41]. The method utilizes 0.6% HS-γ-CD and examines the presence of illicit drugs of abuse by exact mass identification by TOF-MS. The seized drug sample was compared to the previously validated GC-MS method. The exact mass measurements were quite accurate and assisted in drug identification. Each analyzed bath salt contained methylone (*m*/*z* = 207.0892), but one sample also tested for (±)-1-(3,4-dimethyl phenyl)-2-(methylamino)propane-1-one (3,4-DMMC (*m*/*z* = 191.1306) as well. The authors correctly identified that the low injection may be advantageous in CE for forensic analysis. Mikuma et al. [42] used a chemically modified sulfonated capillary, 20 mM sulfated γ-CD, 10 mM formic acid, and pH 2.5 to simultaneously separate all eight amphetamine-type stimulants in 60 min. The developed method was applied to analyze seized methamphetamine samples dissolved in TDI water at a high 1 mg/mL concentration, where (−) ephedrine and (+) pseudoephedrine impurity peaks were also observed. Identifying impurities was much easier to quantitate due to the excellent repeatability of migration time with LOD of about 2 μg/mL for ephedrine and pseudoephedrine. The authors used a chemically bonded capillary to match the migration times in seized methamphetamine samples and authentic standards. Therefore, the chemically modified capillary allowed the accurate repeatability of migration time in CE, which offers an easy and effective way to obtain chiral information on impurities in methamphetamine seizures.

### 5.3. Cell Cultures

**Figure 10:**
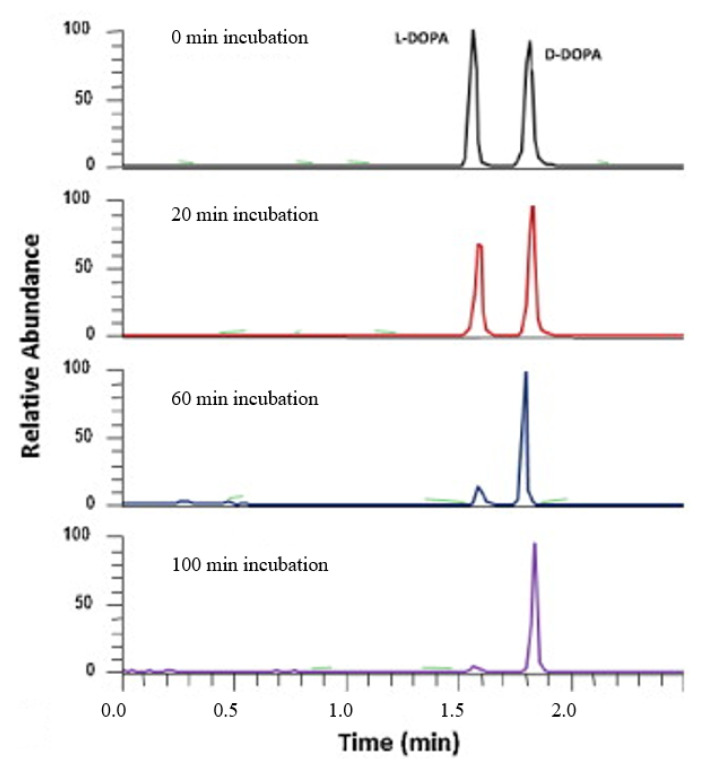
Electropherograms obtained from studying DOPA metabolism in SH-SY5Y neuronal cells. TICs of *m*/*z* 198 from incubation solution injected at different times, and a peak height of *L*-DOPA decreases gradually as incubation time increases while that of co-existing D-DOPA remains unchanged. Both Figure and Figure caption is adapted from Li et al. [38], with permission from Elsevier.

Cell culture analysis is applied to analyze the interaction of drugs and other chemicals with cells. In vitro cell culture is a technique used for understanding the behavior of animal cells or plant cells in a controlled environment, free of systemic variations. The microchip EKC in combination with MS was investigated using 15 mM sulfated β-CD for the determination of three neuroactive AAs glutamic acid, serine, and 3.4-dihydroxyphenylalanine (DOPA)) in less than 2, 2.5, and 3 min, respectively [38]. In particular, DOPA effectively treats Parkinson’s symptoms, as this compound is metabolized by the decarboxylase enzyme in the central nervous system. A series of electropherograms were generated when 50 μM DOPA was analyzed in human SH-SYSY neuronal for enantiomeric quantitation of *L*- and *D*-DOPA every 20 min after incubation. As shown in Figure 10, the *L*-DOPA concentration decreased gradually as incubation time increased while the concentration of coexisting *D*-DOPA remained constant. Thus, the microchip EKC-MS provides conclusive evidence that SH-SYSY cells metabolized *L*-DOPA effectively while leaving *D*-DOPA intact. In a second study, the same research group incubated PC-12 cells with racemic serine by microchip EKC-MS using the same sulfated β-CD as a CS [39]. The authors monitored the serine enantiomeric levels in the media against exposure time. After incubation, both intracellular and extracellular levels of *D*-Ser and *L*-Ser were quantitated. The results suggested that glutamate evokes serine release from PC-12 neuronal cells and the release exhibits a stereochemical preference for *D*-serine over *L*-serine. For example, at 2 min, about 73% of serine released is *D*-serine, and the percentage of *D*-serine decreases as the exposure time increases. At 14 min, more *L*-Ser is present in the media than *D*-Ser. In another study, the PC-12 cells were analyzed by chiral EKC-EKC-MS/MS but without a microchip platform for simultaneous enantiomeric quantification of DOPA and its precursors, tyrosine and phenylalanine [36]. Using a negatively-charged chiral selector, sulfated β-CD, in combination with PFT, avoided potential problems associated with ESI-MS detection. The EKC-MS/MS method was shown to help assess the enantiomeric purity of levodopa, a therapeutic drug for treating Parkinson’s disease, for quality control. In-vitro incubation studies were performed when 1 mM epinine (a dopamine metabolite) was incubated in excess of acetaldehyde under physiological conditions of 50 mM phosphate-buffered saline (pH 7.4 at 37 °C for 3 h [37]. The (*R*/*S*)-*N*-methylsalsolinol ((*R*/*S*)-NMSal) was formed. Additionally, the four isomers of NMSal (i.e., (*R*)-e.e-NMSal (*R*)-e.a-NMSal, (*S*)-e.e-NMSal, and (*S*)-e.a-NMSal) were separated and detected in the incubation solution, suggesting that this Parkinsonian neurotoxin exists in multiple isomeric forms.

### 5.4. Plants and Plant Extracts

**Figure 11:**
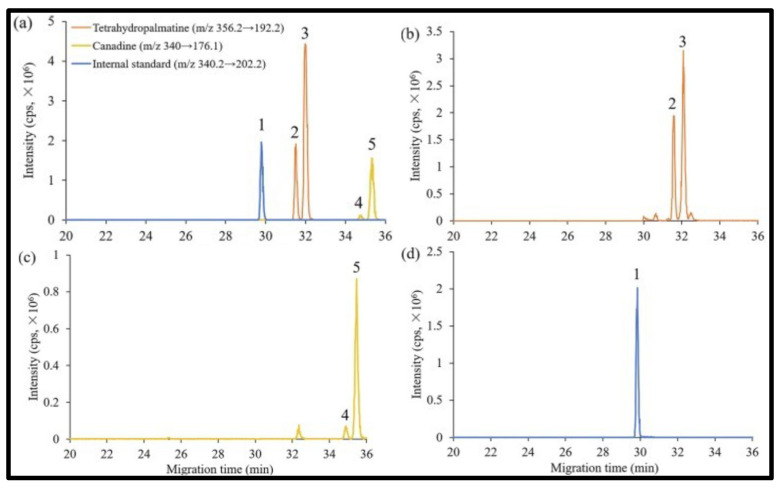
Electropherograms of (**a**) the mixed standard solution (three MRM channels combined) and (**b**–**d**) the Corydalis Rhizoma extract (three MRM channels plotted separately for a clearer view). Peaks: 1. internal standard; 2. *L*-tetrahydropalmatine; 3. *D*-tetrahydropalmatine; 4. (*R*)-canadine; and 5. (*S*)-canadine. Conditions: 100 cm capillary length, 1.68 min partial filling time (at 10 psi), 20 mM HP-β-CD and 20 mM ammonium formate in 10% (*v*/*v*) FA solution as BGE, +24 kV effective separation voltage, 0.1% (*v*/*v*) FA in methanol/water (80:20, *v*/*v*) as chemical modifier with 2 µL/min flowrate. Both Figure and Figure caption were adapted from Yan et al. [33], with permission from Elsevier.

Two studies were reported on plant stereospecific activities by chiral CE-MS [28,33]. To study the bioactivities of jasmonic acid (JA), the EKC-MS/MS using polybrene dextran-coated capillary and PFT was successfully developed for the enantioseparation of racemic JA in the first report [28]. The JA is an essential plant hormone controlling the plant defense signaling system and mediating plant developmental processes, such as root growth, germination, pollen production, and fruit ripening. The naturally occurring JA in the plant is the thermodynamically stable (−)-JA, and its corresponding unstable epimer is (+)-epi-JA. Furthermore, the percentage of JA stereoisomers in the racemic JA standard is 95% for the trans pair of (−)-JA and (+)-JA and 5% for the cis pair of (−)-epi-JA and (+)-epi-JA. The above-mentioned percentages were confirmed by chiral EKC-MS using 20 mM HP-β-CD and peak area normalization. Al-though the stereoisomers of JA are not commercially available, the QTOF-MS allowed the identification of endogenous JA stereoisomers in wounded tobacco leaves. The same samples were spiked at two different levels, and recoveries greater than 74% were obtained. The endogenous JA found in wounded tobacco leaves was just (−)-JA and its corresponding epimer (+)-epi-JA, about 300–500 ng/g FW. Tetrahydropalmatine and tetrahydroberberine (also known as canadine) are two critical bioactive compounds in the Corydalis Rhizoma and Corydalis plant. Their contents are measured as the quality control of Corydalis Rhizoma [33]. The asymmetric carbon in the chemical structure results in two chiral enantiomers for tetrahydropalmatine and tetrahydroberberine, all naturally produced in Corydalis Rhizoma. The chiral separations of four enantiomers in Corydalis Rhizoma extract are shown in Figure 11a. Note there were only a few interfering peaks in the electropherogram of the extract (Figure 11b) despite the complexity of the natural products. The interfering peaks behind *D*-tetrahydropalmatine were the key interfering component, but the effective separation voltage was adjusted to +24 kV to separate this peak (Figure 11b). Notably, the peak of (*R*)-canadine was relatively small compared to that of (*S*)-canadine (Figure 11c). To increase the sensitivity for (*R*)-canadine, the injection time was increased to 12 s without any decrease in the resolution as only 0.3% plug length was injected. Papaverine hydrochloride was selected as the IS because of its structural similarity to tetrahydropalmatine and canadine. Moreover, the migration time of papaverine was slightly shorter than those of the other three analytes, which ensured that IS eluted from the capillary before HP-β-CD.

### 5.5. Miscellaneous

Two further examples of chiral CE-MS analysis include the analysis of AAs in hydrolyzed protein fertilizers (HPFs) [31] and silk textiles [44]. The HPFs contain AAs, oligopeptides, and polypeptides produced primarily from chemical or enzymatic hydrolysis. However, only the enzymatic hydrolysis method does not induce AA racemization and protects the original raw material in its original enantiomeric form. Four commercial HPFs were analyzed for 14 AAs with a resolution greater than 1.0, and LOD of 0.02 μM was achieved by chiral EKC-MS^2^ using 0.75 mM γ-CD in ammonium carbonate as BGE [31]. The samples were obtained through different types of hydrolysis: As expected, HPF#l and HPF#3 obtained by enzymatic hydrolysis contained 98 and 94 per cent enantiomeric excess (ee) of *L*-AAs with *D*-AAs enantiomeric impurity (ei) of less than 2.8 and 1.0%, respectively. On the other hand, HPF#4, obtained by chemical hydrolysis of collagen, provides the highest ei in 14–51% range, whereas the ee of L-AAs ranged from 11–70%. The HPF#2 obtained as a mixture of enzymatic and chemical hydrolysis provided percentage values of ei of D-AAs up to 19.1% and ee of L-AAs to only 61.8%. These results suggest that although there are similar hydrolysis features for HPF#2 and HPF#4, the enzymatic hydrolysis before chemical hydrolysis avoided the conversion substantially to *D*-AAs. All of this data indicates a high potential of the enzymatic method in controlling the quality of hydrolyzed protein fertilizers. A CE-MS method is developed for age estimation of silk textiles by measuring the AAs racemization rates [44]. As expected, less than 100 μg of silk was required, which provided both AA composition and *D*/*L* ratios of aspartic acid. The *L-* to *D*- conversion of aspartic acid was used to date silk textiles (*B. mori*) from several decades to a few thousand years old. The approach proved suitable when 30–60 mM 18-C-6-TCA was used both as a BGE and complexing agent. The 18-C-6-TCA provided excellent chiral resolution for the studied AA enantiomers within reasonable analysis times of 20 min.

## 6. Conclusions

The last 10 years have seen some exciting new developments and applications of chiral CE-MS. The low consumption of exotic chiral selectors, low analyte volume requirement for precious biospecimen, and green technology are three main CE-MS features enabling this hyphenation technique to be one of the best options to carry out enantioselective assays. Four chiral CE-MS modes (EKC-MS, MEKC-MS, CEC-MS, and LECEC or LEKC) are identified in the literature, and their principles of operation are briefly discussed in the Section 2. The data obtained suggest that the various platforms of these CE-MS modes mentioned above will provide a massive amount of information impacting the future development of chiralomics and metabolomics. The sensitivity and robustness are still the choking points of CE-MS. However, practitioners can overcome these sensitivity limitations of CE-MS by developing robust and sensitive nanospray, sheathless interfaces, and jet stream ionization technologies. Additionally, improved column chemistries combined with MS-compatible chiral MoMs and volatile CS for MEKC-MS can be useful for a broad range of enantioselective separations that are repeatable with high sensitivity. In this context, new MS-compatible pseudostationary phases such as volatile chiral surfactant need to be developed. Among the various hyphenation modes, the EKC-MS remains the most popular choice, with ~60% of the published work being performed using this mode of CE-MS. The most popular class of new CS developed in EKC-MS are derivatives of charged CDs (mostly anionic), with an equal number of reports on native and derivatized CDs. One of the main challenges of EKC is the incompatibility of the two popular classes of CS (e.g., CDs and macrocyclic antibiotics)) with MS detection. For example, ion suppression and ionization source contamination lead to a spectral clutter and sensitivity decrease in ESI-MS. Only in a few cases, it is possible to use a low concentration of CDs in partial filling and counter-migration modes to achieve a complete enantioseparation without a loss in MS sensitivity. Chiral ionic liquids (ILs) could pave a new route for enantiomeric recognition. Still, ILs are not reported in EKC-MS, perhaps because they are not commercially available, limiting further developments. Future trends should be redirected to the development of MS-compatible and more enantioselective dual CS for EKC-MS as well as volatile and more versatile MoMs for MEKC-MS. When combined with versatile and efficient preconcentration, these strategies for CS design should allow a higher number of chiral compounds detected at trace levels in various matrices. In contrast to adding a free-floating CS to the BGE in EKC-MS, the chiral CEC–MS mode uses CSP (column) to achieve enantioseparation. The cyclic and linear sugars with multiple chiral centers are the most versatile CS for enantioseparations in CEC. The advantage of chiral CEC-MS over EKC-MS is associated with the CS immobilization on the column, minimizing ionization suppression and chemical noise from the CS, improving MS detection sensitivity. Among the three types of CEC-MS, open tubular appears to be the most straightforward approach for designing chiral CEC and CEC-MS columns. Using chiral monolith or chiral nanoparticles with a high surface area to improve the analyte loading capability is desirable for optimizing enantiomeric excess and MS sensitivity issues. However, there are challenges in making a chiral monolithic and nanoparticle column with a suitable porosity and permeability to fit the need for enantioseparation. Improving the monolithic column fabrication procedure and developing a rationale library of chiral monomers and chiral crosslinkers would help advance the field of monolithic CEC-MS. Systematic studies on the molecularly imprinted polymers and metal-organic framework as stationary phases are warranted and expected to promote the CEC-MS approaches.

Surprisingly, compared to CDs in EKC, only a few CS are introduced for MEKC-MS. Notably, new CS and methodologies publications only comprise 17% of all papers covered. This lower percentage of publication is, perhaps, due to the development of intellectual property protection for new chiral surfactants and MoMs, which prevents authors from publishing research data in open access and traditional journals. Furthermore, the lack of commercialization of volatile chiral surfactants and the cost of production for MS-compatible MoMs may vary depending on the type and availability of the technique used for surfactant synthesis and polymerization. Future trends should be redirected to developing a broad range of MS-compatible chiral micelles for MEKC-MS.

The LECEC, in its various forms (EKC and MEKC), is also an exciting mode that can significantly contribute to the chiral analysis. Inherent in this field is the need to manipulate the metal–ligand ratio in precise ways to improve enantioseparation and ESI-MS detectability of AAs. The development of new chiral surfactants and the chiral columns will offer LE-MEKC and LE-CEC, respectively, to move beyond amino acid separation and detection to other chiral analytes.

Arguably the commercial availability of a dedicated MCE-MS will play a vital role in the ultrafast separation of chiral molecules, primarily due to their extraordinary capacity to monitor the catalytic enantioselective reaction in a high-throughput fashion in cell cultures and incubation solutions. Integration with microchip electrophoresis with MS detector provides the required niche for low-level detection of chiral metabolites in a biospecimen. This need will be pushed even more in the coming years. To summarize, the sheer diversity of ideas, approaches, and applications shown by the authors of this review and the published papers highlighted here over the last 10 years indicates that chiral CE-MS has an exciting decade ahead. Advances that will undoubtedly occur in synthesis, material science, and column engineering will help expand the already impressive application areas of existing chiral CE-MS to enable new separation science applications of chiral molecules with high sensitivity.

## Data Availability

The data presented in this review are available within the article or in the supplementary material.

## Supplementary Materials

The following supporting information can be downloaded at: https://www.mdpi.com/article/10.3390/molecules27134126/s1, Figure S1: Schematic overview of the search strategy, Figure S2: A horizontal bar chart showing journals publishing chiral CE-MS articles over the past 10 years (2010–2020), Figure S3: A pictorial representation of (A) Agilent co-axial sheath liquid CEMS interface, (B) sheathless porous tip interface [65], Figure S4: Schematic of the CE-APPI-MS [66], Figure S5: Schematic diagram of the interface for coupling capillary electrophoresis (CE) with ICP-MS [67], Figure S6: (a) Schematics of partial filling technique in chiral CE-MS using a non-volatile surfactant [68], and (b) schematic representation of the CMT [27], Figure S7: Comparison of (A) low-molecular-mass monomeric (unpolymerized) micelles and (B) high-molecular-mass micelle polymer introduced to ESI-MS [8].

## Abbreviations

The following abbreviations are used in this manuscript:

AMPS	2-acrylamido-2-methyl-1-propane-sulfonic acid
APFO	ammonium perfluorooctanoate (APFO)
ATS	amphetamine-type stimulants
NH_4_OAC	ammonium acetate
NH_4_OH	ammonium hydroxide
APPI	atmospheric pressure photoionization
ATENO	atenolol
α-CD	alpha cyclodextrin
AAs	amino acids
β−CD	beta cyclodextrin
BGE	background electrolyte
[(+)-18-C_6_H_4_-TCA]	(+)-(18- crown-6)-2,3,11,12-tetracarboxylic acid
CD	cyclodextrin
CD-mH	6-mono-deoxy-6[4(2aminoethhyl] imadazolyl]-β-CD
CDMPC-SO_3_	sulfated cellulose dimethylphenylcarbamate
CSP	chiral stationary phase
C and F	centrifugation and filtration
CE-β-CD	carboxyethyl beta cyclodextrin
CEC-MS	capillary electrochromatography mass spectrometry
COUAM	coumachlor
CMT	counter-migration technique
CSF	cerebrospinal fluid
CS	chiral selector
CSP	chiral stationary phase
D and F	dilution and filtration
DGF	drying gas flowrate
DGT	drying gas temperature
DOPA	dihydroxy phenylalanine
DX	duloxetine (DX)
EP	ephedrine
ESI	electrospray ionization
EKC	electrokinetic chromatography
EKC-MS	electrokinetic chromatography mass spectrometry
3-FMC	fluoromethcathinone
FITC	fluorescein isothiocyanate
FLEC	(+)-1-(9-fluorenyl)ethyl chloroformate ((+)-FLEC)
FMOC	fluorenylmethoxycarbonyl
FFT	full filling technique
FASS,	field amplified sample stacking
γ-CD	gamma cyclodextrin
GMA-β-CD-VBTA	glycidyl methacrylate-bonded β-CD
HS-γ-CD	highly sulfated gamma cyclodextrin
HP-β-CD	hydroxypropyl-β-CD;
ICP-MS	inductively coupled plasma mass spectrometry
ITP	isotachophoresis
IS	internal standard
IT-MS	ion-trap mass spectrometer
JA	jasmonic acid
LIF	laser-induced fluorescence
LE-EKC	ligand exchange electrokinetic chromatography
LOD	limit of detection
LOQ	limit of quantitation
LLE	liquid-liquid extraction
LVSS	large volume sample stacking
MAX	mixed mode anion exchange
MCE-MS	microchip electrophoresis mass spectrometry
MEKC-MS	micellar electrokinetic chromatography mass spectrometry
METOP	metoprolol
MDOPA	methyldopa
MoMs	molecular micelles
MSS	micelle to solvent stacking
*N*-DVX	*N*-desmethylvenlafaxine
NMSal	*N*-methylsalsolinol
OT-CEC	open tubular capillary electrochromatography
O-DVX	*O*-desmethylvenlafaxine
OH-WAR	hydroxyl warfarin
PFT	partial filling technique
poly-α-*D*-SOGP	polysodium N-octenylenyl-alpha-D-glucopyranoside 4,6-hydrogen phosphate
poly-α-*D*-SUGP	polysodium *N*-undecenylenyl-alpha-D-glucopyranoside 4,6-hydrogen phosphate
poly-α-*D*-SOGS	polysodium *N*-octenyl-alpha-D-glucopyranoside 6-hydrogen sulfate
poly-α-*D*-SUGS	polysodium *N*-undecenynl-alpha-D-glucopyranoside 6-hydrogen sulfate
poly(*L*-SUCL)	polysodium undeceoxy carbonyl leucinate
poly(*L*-SULV)	polysodium undecenyl valinate
poly(*L*-SULA)	polysodium undecenoyl *L,L*-leucyl alaninate
poly(*L*-SULV)	polysodium undecenoyl *L,L*-leucyl valaninate
PEP	pseudoephedrine
PSP	pseudostationary phase
QQQ-MS	triple quadrupole mass analyzer
Q-TOF-MS	quadrupole time of flight mass spectrometer
SAADCL	sodium 10-acryl-amidodecenoxy carbonyl-L-leucinate
SLE	solid liquid extraction
SPE	solid
S-β-CD	sulfated beta cyclodextrin
SBE-β-CD	sulfobutyl ether beta cyclodextin
SL	sheath liquid
SQ	single quadrupole mass analyzer
UV/DAD	ultraviolet/diode array
VC	vancomycin chloride
VX	venlafaxine
WAR	warfarin
