# Supplementary material for: Capillary Electrophoresis Mass Spectrometry: Developments and Applications for Enantioselective Analysis from 2011–2020
Source: Molecules. 2022 Jun 27;27(13):4126. doi: 10.3390/molecules27134126 (PMC9268241; doi:10.3390/molecules27134126)
Supplement: Supplementary file 1 [file molecules-27-04126-s001.zip › molecules-1567993-supplementary.pdf]

Capillary electrophoresis mass spectrometry: Developments and applications for enantioselective analysis from 2011-2020

Supplementary Information

***Shahab A. Shamsi and Ferdoushi Akter**
Department of Chemistry
Georgia State University
Atlanta, GA 30303

***Corresponding Author: Email: sshamsi@gsu.edu**

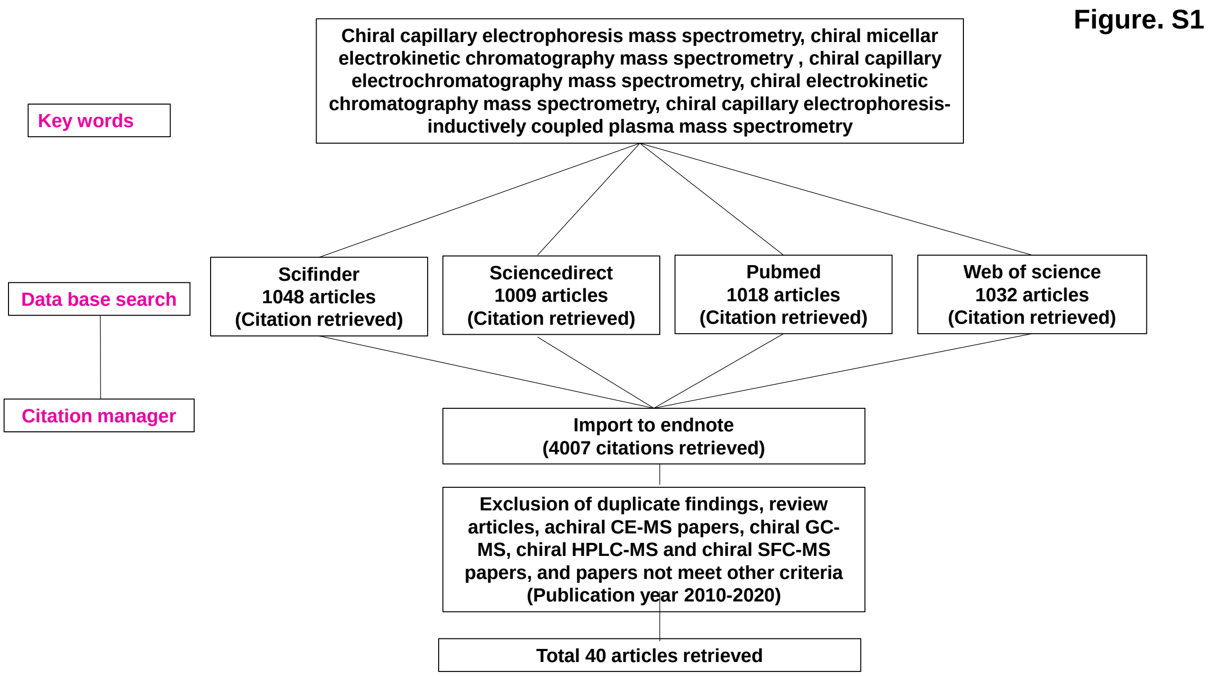

Figure S1. Schematic overview of the search strategy.

Figure. S2

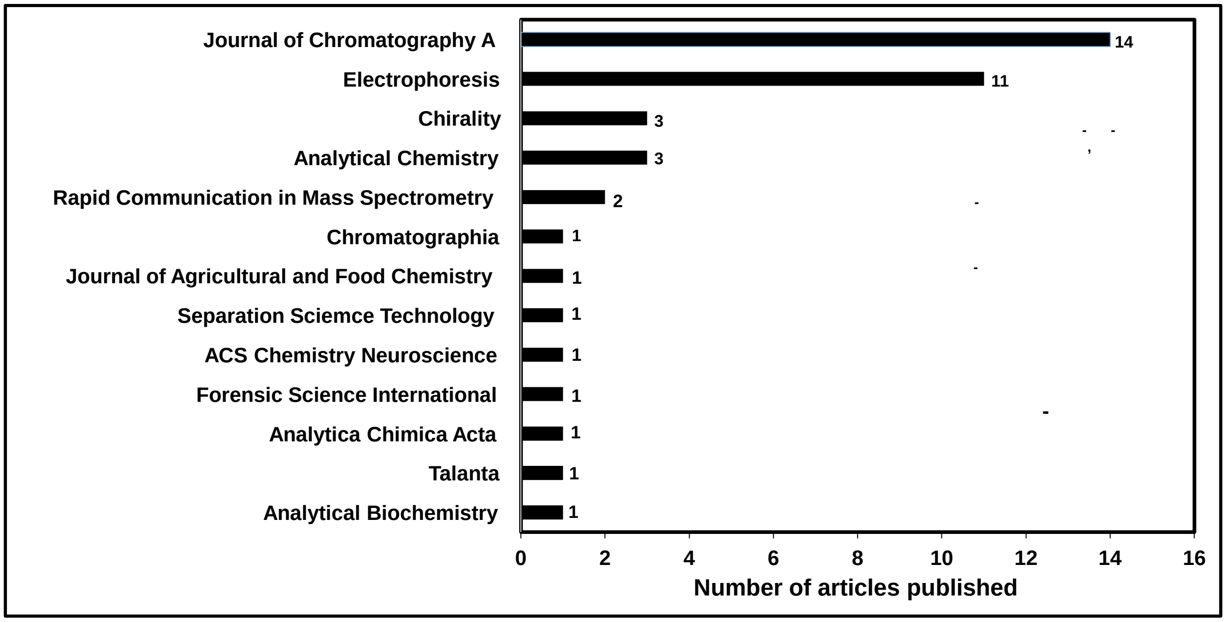

Figure S2. A horizontal bar chart showing journals publishing chiral CE-MS articles over the past ten years)2011-2020(.

Figure. S3

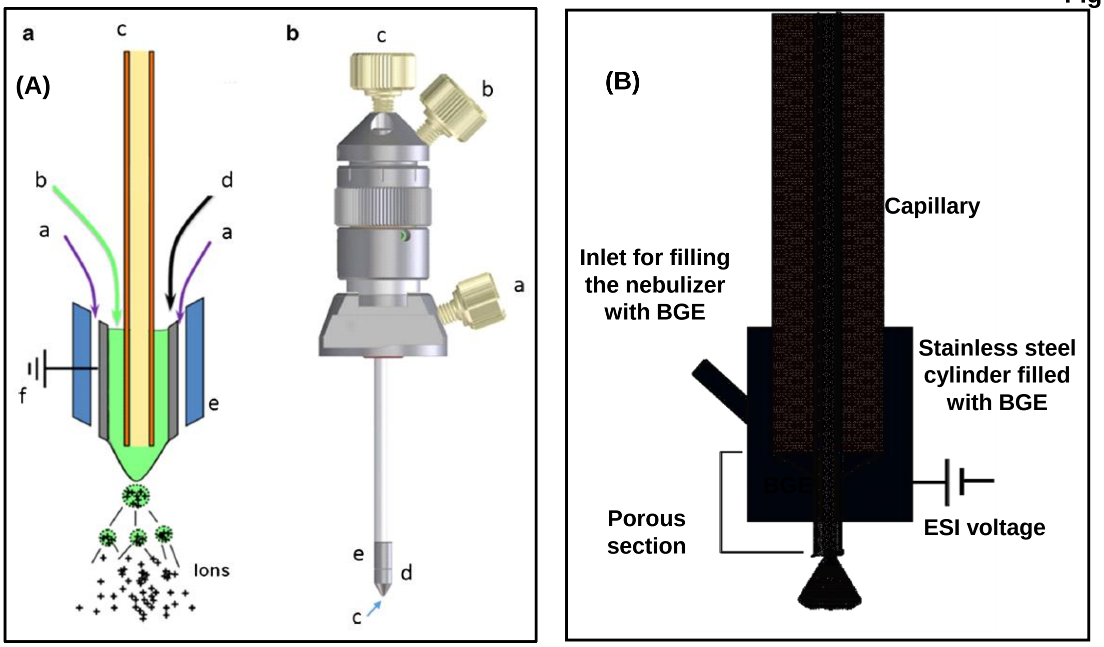

Figure S3. A pictorial representation of (A) Agilent co-axial sheath liquid CEMS interface: a nebulizing gas, b Sheath liquid, c CE-capillary with BGE, d Stainless steel spray needle 0.4 mm i.d., 0.5 mm o.d., e outer tube, f ground connection. b Engineering sketch of the coaxial sheath liquid CEMS interface (graphics courtesy with permission from Agilent Technologies), and (B) sheathless porous tip interface. A scheme of the porous tip (sheathless) interface, initially developed by Moini, is depicted. Figure 6B is adapted from Frasl et al. [65] with permission from American Chemical Society.

Figure. S4

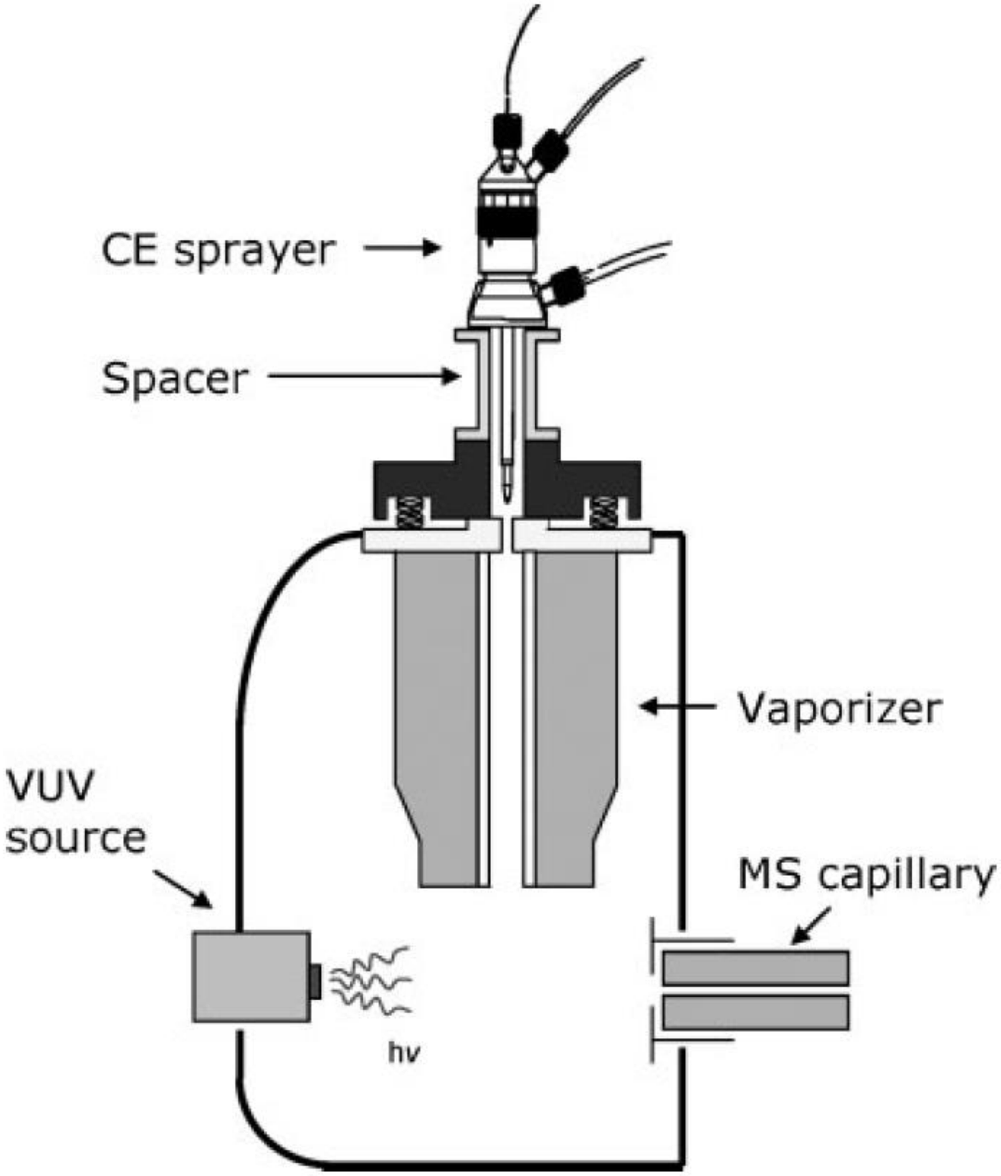

Figure S4. Schematic of the CE-APPI-MS system comprising CE sprayer, spacer, vaporizer, and krypton discharge lamp (VUV source). Reproduced from [66] with permission.

Figure. S5

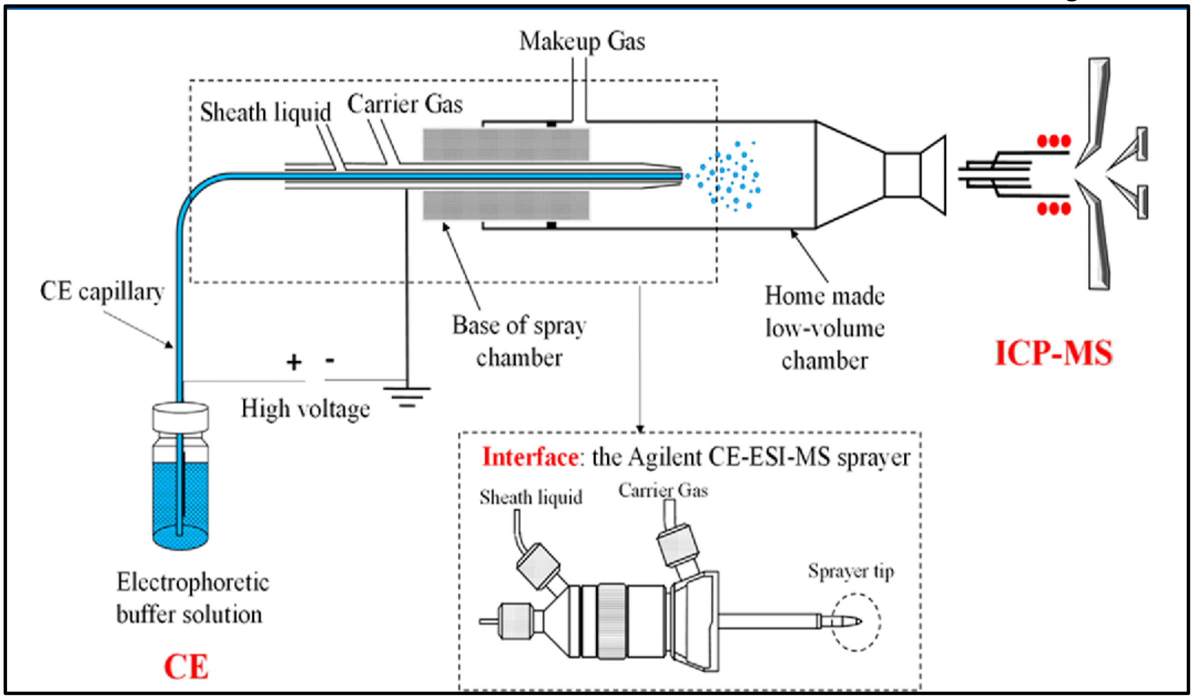

Figure S5. Schematic diagram of the interface for coupling capillary electrophoresis (CE) with inductively coupled plasma mass spectrometry (ICP-MS). Reproduced from [67] with permission from the American Chemical Society, copyright 2014.

Figure S6

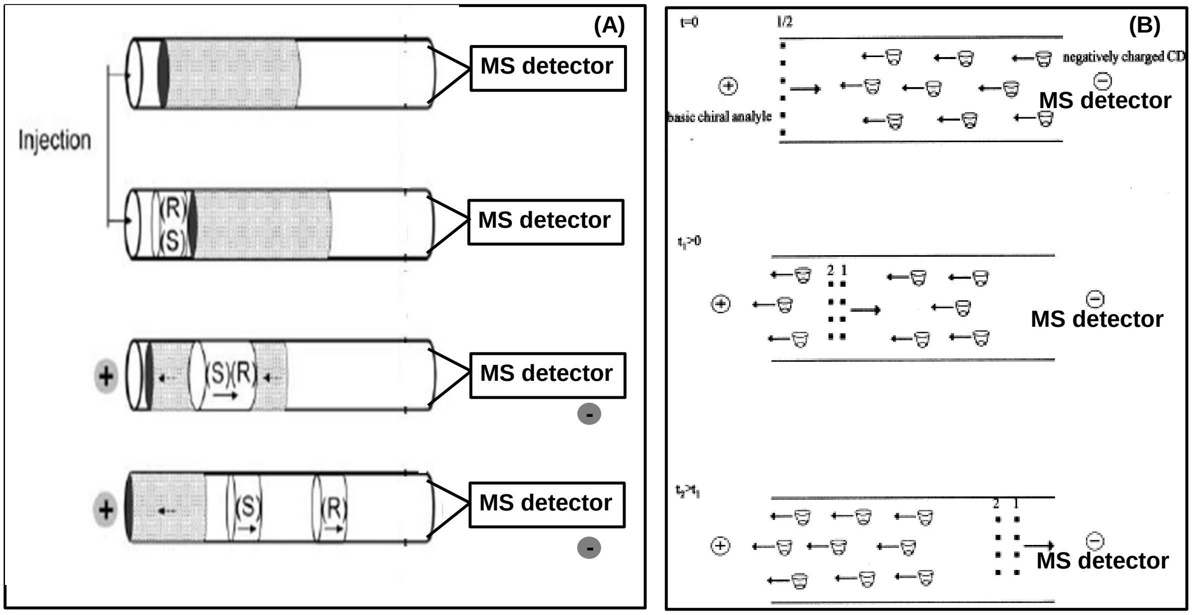

Figure S6. (a) Schematics of partial filling technique in chiral CE-MS using a non-volatile surfactant, reprinted with permission from [69] and (b) Schematic representation of the counter-migration principle of a basic analyte and a negatively charged CD, reproduced from [69] with permission.

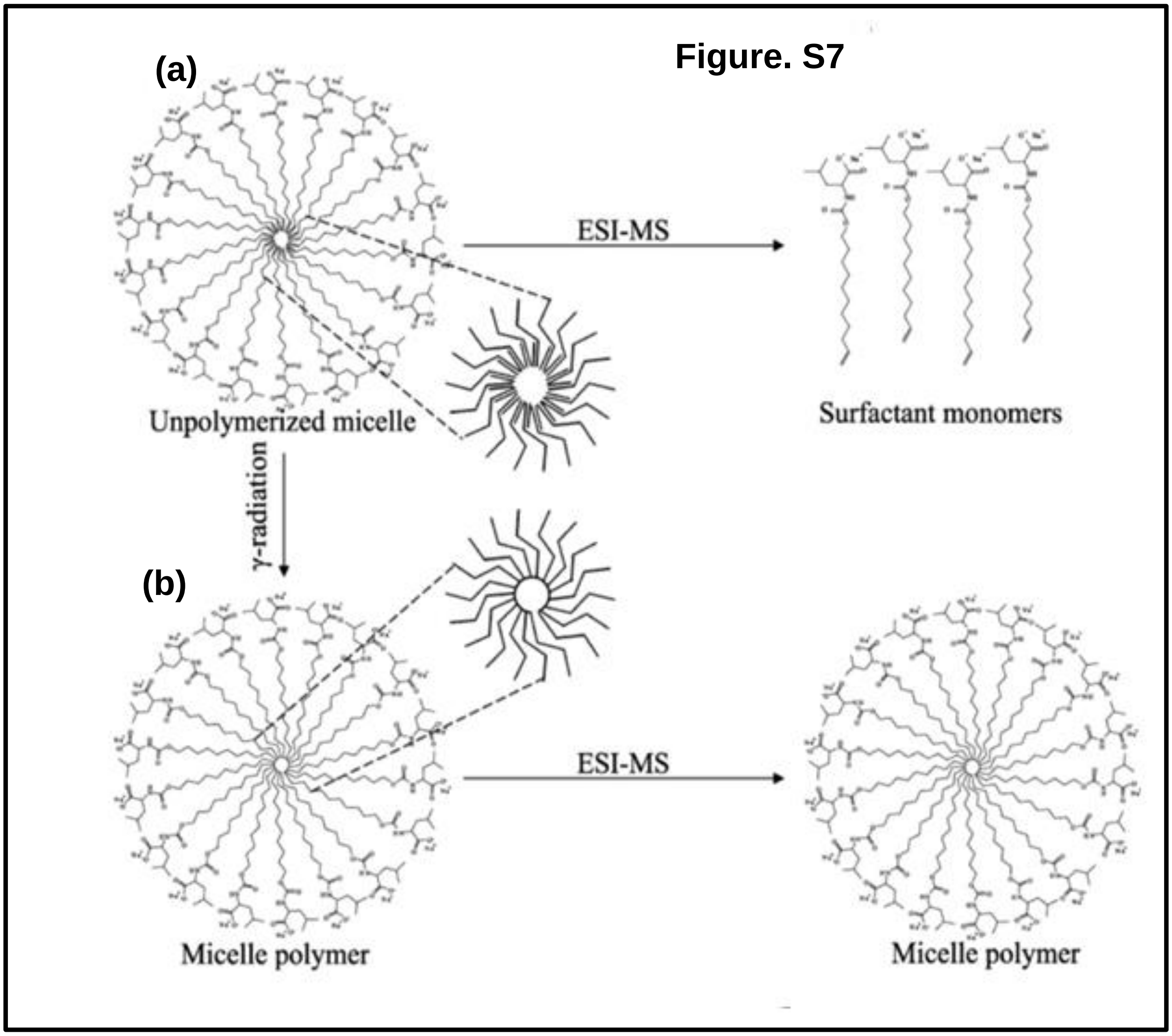

Figure S7. Comparison of (A) low-molecular-mass monomeric (unpolymerized) micelles and (B) high-molecular-mass micelle polymer introduced to ESI-MS, reproduced from [8] with permission from American Chemical Society.
